# Phenotypic characterization and seed viability test in *ex-situ* conserved Ethiopian cultivated barley (*Hordeum vulgare* L.) landraces

**DOI:** 10.1186/s12870-023-04628-7

**Published:** 2023-12-04

**Authors:** Fekadu Gadissa, Temesgen Bedassa Gudeta

**Affiliations:** College of Natural and Computational Sciences, Biology Department, Madda Walabu University, P.O. Box 247, Bale Robe, Ethiopia

**Keywords:** Barley, Diversity, Landraces, Performance evaluation, Phenotypic characters

## Abstract

**Background:**

Nowadays, most of the Ethiopian barley landraces had been lost from farmer’s field and exclusively found *ex-situ* conserved at the Ethiopian Biodiversity Institute (EBI). Those *ex-situ* conserved are generally believed to be representative of the original population and possess high genetic diversity and important unique genes that are useful for tackling the various biotic and abiotic stresses in the face of the current climate change. Thus, this research was aimed at testing the performance of 150 *ex-situ* conserved landraces that had been collected from Arsi and Bale highlands, Southeastern Ethiopia. The landraces were tested at multiple test locations over two years (2021 and 2022).

**Results:**

All the tested landraces showed a good germination rate regardless of their long storage duration. In addition, performance of all the qualitative traits revealed a varying frequency for each character state. For example, most of the accessions (51.3%) had six kernel row numbers (KRN). All the remaining accessions had two rows (28.7%) and irregular KRN with variable lateral florets (20%). Likewise, some of the quantitative traits considered showed a significant variation among the landraces. However, there observed a significant variation for all the interaction effects in some of the traits considered signifying the importance of considering environment effects while targeting genetic selection and improvement of *ex-situ* conserved germplasms. The phenotypic coefficients of variation (PCV) were considerably high to medium in most of the traits considered including seed yield per hectare (SYPH) but with no associated higher genotypic coefficients of variation (GCV). Moreover, all the traits showed a far greater phenotypic coefficient of variation (PCV) to that of genotypic coefficients of variation (GCV) once again suggesting the pronounced effect of environmental factors to the variation. This was far supported by the significantly higher absolute magnitudes in phenotypic correlation compared to their corresponding genotypic correlation in most of the traits. Low estimates of heritability and genetic advance observed in all the traits considered except seed yield per hectare indicate importance of the trait for selection in Ethiopian barley improvement programs. Clustering patterns of the accessions, in narrow sense, revealed the existence of low divergence among the samples.

**Conclusion:**

Ethiopian barley landraces are promising candidates for further yield improvement and conservation. However, further regular testing and screening should be conducted for the *ex-situ* conserved landraces because of the current erratic climate change. In addition, more robust molecular marker systems could be used to clearly reveal the extents of genetic diversity and to facilitate the breeding and conservation of Ethiopian barley landraces.

**Supplementary Information:**

The online version contains supplementary material available at 10.1186/s12870-023-04628-7.

## Introduction

Ethiopia is one of the world’s richest genetic resource centers for various food crops including cultivated barley [1]. The country is home for several indigenous food crops, which purely constitute landraces that have been maintained over centuries by farmers, mainly through traditional cultivation systems. They are genetic resources that are believed to have considerable breeding value due to their co-adapted gene complexes with tolerance or adaptation to diseases and environmental constraints [2]. In addition, they are useful in breeding for marginal conditions [3] as they offer genes responsible for a more stable yield over a wide range of environmental conditions [4, 5].

Cultivated barley (*Hordeum vulgare* L.) is among the top genetic resources for which the country is known to be the secondary center of diversity, according to Vavilov [1] and claimed to be the center of origin, according to Bekele [6] and Negassa [7]. Until recently, its cultivation is purely traditional and hence, exists largely in landrace form [8]. Moreover, it is among the top neglected cereals regardless of its huge potential for subsidizing household food security [9], and supporting national and international breeding programs targeting improved adaptation potential to enhance resilience to drought, diseases and other biotic and abiotic crises [10]. In this regard, since its start in the 1950s, barley research has gained several successes. So far, more than 36 improved food barley varieties with different unique important characters and breeding objectives have been released [11].

However, in recent days, the cultivation of Ethiopian barley landraces is declining to the extent of total genetic loss because of the preference for and replacement with a limited number of modern, genetically uniform cultivars or other crops suited for high input agriculture [12, 13]. For instance, some earlier morphotypes such as hulled barley, smooth-awned types, hull-less types, many naked and some rare covered forms are no longer found in Ethiopia [12]. Some are only found *ex situ* conserved at the Ethiopian Biodiversity Institute (EBI), Addis Ababa, Ethiopia, which has been extensively collecting and documenting several landraces from the widely producing corridors of the country. So far, the institute has documented more than 17,000 collections from the major barley-growing regions and zones of the country, including the Arsi-Bale highlands [14]. Most of those collections were kept at the Ethiopian Biodiversity Institute (EBI) or former Gene Bank for several decades as an *ex-situ* conservation effort and are completely absent from the farmers’ field. In addition, the landraces have not yet been taken back to the farmers’ fields to be evaluated for their important agronomic traits, an important step for further large-scale utilization and conservation efforts of the available genetic resources [15]. As a consequence, there is a paucity of valuable up-to-date information on the performance of the Ethiopian barley landraces under the currently changing climatic and soil conditions. However, such information is essential for parental selection in order to develop best-performing, highly productive and good quality varieties, as well as for production [16–19], and for planning efficient germplasm conservation and utilization strategies [20, 21].

Therefore, the present study was initiated to assess the overall performance of *ex situ* conserved barley landraces deposited at the Ethiopian Biodiversity Institute (EBI) for a long period so as to generate comprehensive and well-organized information on the extent of their germination rate and genetic diversity using agro-morphological traits and to study the relationship between yield-related traits. The information generated would be used as an important input for further improvement and conservation programs. In addition, it could be used as baseline information to develop national and international plans regarding the characterization and utilization of *ex situ* conserved landraces**.**

## Results

### Germination tests

Germination performance, including germination percentage, mean germination time (MGT), and germination rate per day for the evaluated *ex-situ* conserved barley accessions is presented under Table 1. Most of the accessions showed promising germination percentage, where the highest score of 100% was recorded in three accessions, namely EBL025 (3246), EBL052 (3273), and EBL133 (212844), all of which were collected 39–44 years ago (the first two in 1979 and the third in 1984). The smallest germination percentage (46.2%) was recorded in EBL089 (4462) that was of recent collection (1980) as compared to several other accessions. The range and mean germination time across the accessions showed very minimum variation, the smallest being 9.31 in EBL023 (1723) and the largest being 13.99 in EBL136 (212847). These two extreme accessions had been *ex-situ* conserved for nearly 40 years at the EBI. Likewise, the germination rate showed a minor variation across the accessions where the highest (0.15) was exhibited by EBL124 or 64245 and the smallest (0.07) in EBL083 or 3833 and EBL146 or 215373.

**Table 1:** Summary on the germination performance of the *ex-situ* conserved Ethiopian barley landraces considered in the present study

Year of collection	Number and entry code of accessions	Percent (%) Germination	Mean Germination Time (MGT)	Germination per day
1964	**22** (*EBL001—EBL022*)	48.2—98.9	10.03—13.21	0.08—0.12
1978	**16** (*EBL023—EBL038*)	46.7 – 100.0	9.31—13.67	0.08—0.14
1979	**46** (*EBL039—EBL092*)	48.7 – 100.0	9.89—13.92	0.08—0.13
1980	**8** (*EBL084—EBL091*)	46.2—66.7	10.82—12.67	0.08—0.09
1981	**32** (*EBL093—EBL124*)	46.7—99.2	10.03—13.23	0.08—0.15
1982	**6** (*EBL125—EBL130*)	56.7—97.0	10.03—12.50	0.09—0.12
1984	**9** (*EBL131—EBL139*)	53.3 – 100.0	10.73—13.99	0.08—0.14
1985	**11** (*EBL140—EBL150*)	46.7—78.6	10.50—13.06	0.07—0.11

### Qualitative morphological traits

Performance of the accessions assessed in terms of the eleven qualitative traits is presented under Table 2. In this regard, most of the accessions (51.34%) had six kernel row numbers (KRN). All the remaining accessions had two rows (28.7%) and irregular KRN with variable lateral florets (20%). Long awns and awn less accessions accounted for nearly equal proportions of the six-KRN accessions at 26.7% and 24.7%, respectively. Of the two-rowed barley accessions, those with lateral florets accounting for 19% and were the most common type as compared to the two-rowed deficient type, which accounted for 9%. In terms of kernel covering (KC), the majority (65%) had covered grains, while 20% and 15% of the accessions studied, respectively represented naked and semi-covered grains. Among all lema color (LC) types, yellow (47%) was found to be the most common, and this was followed by black/gray (25%) lema color, while tan/red (13%) and purple (15%) were fewer. The distribution of lemma awn barb (LAB) was nearly proportionate across the accessions, with specific distributions containing smooth (35%), intermediate (32%), and rough (33%). However, the distribution of lemma type varied across the accessions, where most had lemma teeth (38%) and lemma hair (37%), while the remaining (25%) had no lemma teeth.

**Table 2:** Characters, frequency and percent coverage of qualitative morphological traits in the tested 149 *ex situ* conserved Ethiopian barley accessions and one local check

Character or variable	Trait	Score	No of accessions (freq.)	% accessions possessing a phenotype	Trait	Phenological traits	Score	No of accessions (freq.)	% accessions possessing a phenotype
Kernel row number (KRN)	Two rowed, large/small sterile lateral florets	1	29	19.3	Awn colour (AC)	White	1	53	35.3
	Two rowed, deficient	2	14	9.3		Yellow	2	31	20.7
	Irregular, variable lateral florates	3	30	20.0		Brown	3	24	16.0
	Six rowed, awnless	4	40	26.7		Reddish	4	19	12.7
	Six rowed, long awns	5	37	24.7		Black	5	23	15.3
Long Kernel covering (KC)	Naked grain	1	30	20.0	Spike density (SD)	Lax	1	27	18.0
	Semi-covered grain	2	22	14.7		Intermediate	2	67	44.7
	Covered grain	3	98	65.3		Dense	3	56	37.3
Lemma/Kernel color (LC)	Yellow	1	71	47.3	Length of rachila hair (LRH)	Short	1	59	39.3
	Tan/red	2	19	12.7		long	2	91	60.7
	Purple	3	22	14.7	Stem Pigmentation (SP)	Green	1	87	58.0
	Black/grey	4	38	25.3		Purple (basal only)	2	33	22.0
Lemma awn barb (LAB)	Smooth	1	52	34.7		Purple (half or more)	3	30	20.0
	Intermediate (small barbs)	2	48	32.0	Glumes colour (GLC)	White	1	70	46.7
	Rough	3	50	33.3		Yellow	2	36	24.0
Lemma type (LT)	No lemma teeth	1	38	25.3		Brown	3	24	16.0
	Lemma teeth	2	57	38.0		Balck	4	20	13.3
	Lemma hair	3	55	36.7					
Growth habit (GH)	Prostrate	1	21	14.0					
	Intermediate	2	32	21.3					
	Erect	3	97	64.7					

With regards to growth habit (GH), most of the accessions (65%) were erect, followed by intermediate (21%) and prostate (14%). Accessions with white (35%) awn color were more frequent as compared to those with yellow (21%), brown (16%), black (15%), and reddish colors (13%). Regarding spike density (SD), a large number of accessions had an intermediate density (45%), which was followed by dense (37.3%), and lax (18.0%). Most of the accessions (61%) had long rachilar hair (LH), while some 39% had short lachilar hair. A large number of the accessions had green stem pigmentation (58%), which was followed by purple (basal only) (22%), and purple half or many (20%). The frequency distribution of the accessions with regard to glumes color (GLC) indicated that white (47%) was the most frequent, and was followed by yellow (24%), brown (16%), and black (13%), which was the least frequent.

### Quantitative morphological traits

#### Descriptive statistics

Descriptive statistics of the quantitative traits measured in the barley accessions are presented in Table 3. In general, the *ex-situ* conserved barley landraces showed a wide range of variability, as evidenced by the wide range for most of the quantitative traits. Accordingly, seed yield per hectare (SYPH) showed the widest range (3,210.8 kg/ha) with an average mean performance value of 2,249.9 ± 9.81 kg/ha. This was followed by plant height (PH), which had a combined average performance of 112.0 ± 0.34 cm with a range of 112.0. The accessions performed well for disease traits such as recovery rate per stand (RPS), with a combined mean performance of 82.0 ± 0.23 and range of 50.00 and net blotch (NB) with mean performance of 75.9 ± 0.14 and range unit of 44.

**Table 3:** Range and mean of the 18 quantitative traits combined over the three experimental locations and two seasons (St Er = standard error; SD = standard deviation)

Traits^a^	Max	Min	Range	Mean	St Er	*SD*
DTE	11.0	6.0	5.0	8.1	0.02	8.08
DTH	97.0	56.0	41.0	74.9	0.16	7.06
DTM	138.0	99.0	39.0	119.3	0.21	9.01
PH	146.0	34.6	112.0	97.5	0.34	14.43
LW	2.1	0.3	1.8	1.3	0.02	0.71
FLL	37.0	8.0	29.0	21.2	0.09	4.19
LN	6.0	3.0	2.9	5.3	0.02	0.96
SLA	39.0	7.2	31.8	18.6	0.31	13.31
NGPP	49.0	11.9	37.2	31.9	0.14	5.97
SL	15.7	3.6	12.1	9.3	0.09	3.71
AL	20.0	6.3	13.7	11.4	0.04	1.71
EFT	65.0	3.0	62.0	23.2	0.24	10.22
TSW	96.0	27.6	29.1	72.4	0.11	4.57
SYPH	4566.8	1355.9	3210.8	2249.9	9.81	416.12
NB	99.0	55.0	44.0	75.9	0.14	5.72
LR	32.0	4.0	28.0	13.6	0.09	3.63
Inf	64.0	6.0	58.0	18.4	0.23	9.88
RPS	105.0	40.0	65.0	82.9	0.23	9.78

^a^*DTE* Days to emergency, *DTH* Days to heading, *DTM* Days to maturity, *PH* Plant height, *LW* Leaf width, *FLL* Flag leaf length, *LN* Number of leaves per plant, *SLA* Single leaf area, *NGPP* Number of grains per plant, *SL* Spike length, *AL* Awn length, *EFT* Effective fertile tiller, *TSW* Thousand seeds weight, *SYPH* Seed yield per hectare, *NB* Net blotch, *LR* Leaf rust, *Inf* Rate of infestation, *RPS* Recovery per stand, SD Standard deviation

### Analysis of variance (ANOVA)

The analysis of variance for the quantitative traits computed using the data combined over the three locations and two seasons (years) is presented in Table 4. Most of the quantitative traits showed highly significant (*P* < *0.01*) variation over years, locations, and year-by-location interactions. Likewise, the mean square values in most traits (eleven out of the total eighteen) showed a highly significant (*P* < *0.01*) or significant (*P* < *0.05*) variation among the accessions, accession-by-year and accession-by-location interactions. On the other hand, mean square values of only some traits (six out of the total eighteen) showed a significant (*P* < *0.05*) variation for year-by-location-by-accession interactions.

**Table 4:** Combined analysis of variance (ANOVA) for the 18 quantitative traits considered

Traits*	Year ***(1)***	Loc ***(2)***	Acc ***(149)***	Year*Loc ***(2)***	Year*Acc (***149)***	Loc*Acc ***(298)***	Year*Loc*Acc ***(298)***	MSE ***(894)***	R^2^	CV
DTE	185.0***	2.2*	0.5*	299.8***	0.4*	0.5	0.5**	0.5	0.74	8.66
DTH	43.1	22,515.8***	23.8	46.2	25.4	32.4***	30.2	29.6	0.73	7.23
DTM	2010.8***	29,050.4***	67.8***	1517.9***	40.7	50.8**	37.7	37.4	0.76	5.13
PH	24,401.7***	69,551.2***	136.9**	12,529.9***	117.4***	143.1	108.1	136.9	0.68	11.99
LW	0.7	13.8	0.5	5.1***	0.5	0.5	0.5*	8.3	0.50	10.23
FLL	56.4**	598.1***	15.7	218.9***	15.1	13.6	13.7	13.2	0.63	17.08
LN	2.4***	197.1***	0.7	137.3***	0.6	0.6	0.6	0.6	0.71	14.95
SLA	1918.2***	3107.1***	27.3	2357.8***	28.5**	27.5	24.3	28.7	0.64	23.53
NGPP	266.1**	13.3	30.6***	8.0	29.5*	35.5**	35.2	36.6	0.49	18.59
SL	8.4**	241.3***	5.1	67.6***	3.1	3.1*	13.4	2.9	0.59	17.99
AL	4.5	260.3***	2.9	2.3	1.7	2.6	2.8	2.5	0.60	13.75
EFT	318.5*	13,670.7***	84.3*	543.1**	91.5*	74.9**	73.7***	70.6	0.63	36.27
TSW	333.6	329.7	557.1**	1.1	532.4**	544.1**	553.8**	523.7	0.53	12.73
SYPH	1497.5	166,183.7*	174,191.0*	81.9	181,308.6*	177,236.9*	167,980.5*	168,979.8	0.52	18.13
NB	467.0**	192.6**	38.1***	132.3*	40.8**	38.2**	37.4**	37.8	0.51	8.09
LR	0.7*	4.8*	13.5*	11.1	11.9*	14.8***	14.1	13.3	0.52	26.90
Inf	64.9**	1.5**	70.3**	3.8	74.4***	74.1*	81.3	76.7	0.50	47.57
RPS	47.0*	26.1***	83.6***	138.7	101.3*	85.4***	93.5	92.2	0.50	11.58

Numbers in brackets under the first row represent degrees of freedom (df)

*Loc* Location, *Rep* Replication, *Trt* treatment (accessions), *Acc* Accessions, Year*Loc Year-Location interaction, Year*Trt Year-Treatment interaction, Loc*Trt Location-Treatment interaction, Year*Loc*Trt Year-Location-Treatment interactions, Rep(Year*Loc) Replication within year-location interactions, *MSE* Mean square error, *R*^*2*^ Coefficient of genetic determination, *CV* Coefficients of variation

^*^significant at *p* < 0.05

^**^highly significant at *p* < 0.01

^***^highly significant at *p* < 0.001

^*^Details of the traits used is presented under Table 3

All of the quantitative traits showed a coefficient of genetic determination (R^2^) nearly greater than or equal to 0.5 with greater scores in days to maturity (DTM) and days to emergence (DTE) (each 0.8 and 0.7, respectively). Similarly, the coefficients of variation (CV) were moderate for most of the traits and within the acceptable range except in effective fertile tiller (EFT) and rate of infestation (Inf) that had 47.6 and 55.6, respectively (Table 4).

### Analysis of components of variance

Estimates of variance components of the quantitative traits computed using combined data is presented under Table 5. Estimate of both phenotypic (δ^2^_p_) and genotypic (δ^2^_g_) variances showed a wide range of variation (0.0 and 0.1 in leaf width or LW to 1709.9 and 174529.2 in seed yield per hectare or SYPH). Similarly, estimate of variance due to genotype-year (σ^2^gy), genotype-location (σ^2^gl), and genotype-year-location (σ^2^gyl) interactions showed a wide range of variation when the traits are considered all together. However, the variations in all the traits showed narrow range when disregarding seed yield per hectare (SYPH). Estimate of error (environment) variance (δ^2^_e_) also revealed a wide range (0.1 in leaf width or LW to 166971.3 in seed yield per hectare or SYPH).

**Table 5:** Estimate of variance components computed using data combined over the three test locations and two test seasons

Traits^a^	Mean	σ^2^g	σ^2^gy	σ^2^gl	σ^2^gyl	σ^2^e	σ^2^P	PCV	GCV	GECV	H^2^%	GA	GAM
DTE	8.1	0.01	0.00	0.00	0.01	0.35	0.37	7.53	1.24	1.24	2.70	0.03	0.42
DTH	74.8	10.02	0.78	0.03	1.04	24.15	26.02	6.82	0.19	1.36	38.51	4.04	0.05
DTM	119.3	1.55	0.13	5.23	1.50	42.74	5.15	6.00	1.04	1.03	30.10	1.41	1.18
PH	97.3	0.16	0.12	6.25	12.19	127.92	146.64	12.44	0.41	3.59	0.11	0.03	0.03
LW	1.2	0.00	0.00	0.00	0.00	0.05	0.05	19.11	0.00	0.00	0.00	0.00	0.00
FLL	21.2	0.19	0.53	0.20	0.68	13.71	15.31	18.49	2.06	3.90	1.24	0.10	0.47
LN	5.1	0.01	0.02	0.01	0.01	0.39	0.44	12.96	1.95	1.95	2.27	0.03	0.61
SLA	18.7	0.23	0.79	0.34	2.08	25.71	29.15	28.95	2.57	7.73	0.79	0.09	0.47
NGPP	32.4	0.13	0.89	0.04	0.08	36.75	37.89	18.99	1.11	0.87	0.34	0.04	0.13
SL	9.3	0.03	0.00	0.08	0.08	2.91	3.10	18.99	1.87	3.05	0.97	0.04	0.38
AL	11.4	0.12	0.15	0.05	0.13	2.33	2.78	14.66	3.05	3.17	4.32	0.15	1.30
EFT	23.1	1.24	3.43	3.13	3.19	77.31	88.30	40.66	4.82	7.73	1.40	0.27	1.18
TSW	39.4	10.12	0.07	0.15	0.88	21.36	22.58	12.06	0.88	2.38	44.82	4.38	11.011
SYPH	2254.5	1709.90	2831.00	2325.60	691.41	166971.30	17452.21	18.53	1.83	1.17	9.80	26.62	1.18
NB	75.5	0.34	0.08	0.09	0.41	32.51	33.43	7.66	0.77	0.85	1.02	0.12	0.16
LR	12.5	0.02	0.19	0.34	0.28	13.31	14.14	30.06	1.13	4.23	0.14	0.01	0.09
Inf	17.9	1.14	1.09	5.59	3.99	99.39	111.20	58.85	5.96	11.15	1.03	0.22	1.24
RPS	82.0	1.06	0.85	4.94	3.28	97.36	107.49	12.64	1.26	2.21	0.99	0.21	0.26

Genotype (g) was used in terms of treatments (accessions)

*σ*^*2*^*gy* variance of genotype-year interaction, *σ*^*2*^*gl* variance of genotype-location interaction, *σ*^*2*^*gyl* variance of genotype-location-year interactions, *σ*^*2*^*g* genotypic variance, *σ*^*2*^*p* Phenotypic variance, *σ*^*2*^*e* variance of error, *H*^*2*^ heritability in broad sense, *GA* genetic advance, *GAM* genetic advance percentage of mean

^a^Description of the traits is presented under Table 3

Likewise, estimates of both phenotypic (PCV) and genotypic coefficients of variations (GCV) showed a wide range of variations (PCV = 6.0 in days to maturity or DTM to 58.82 in rate of infestation or Inf; GCV** = **0.0 in leaf width or LW to 5.9 in infestation or Inf). All the traits considered showed a lower estimate of GCV, the highest score being 5.9 in rate of infestation (Inf). On the other hand, four traits such as rate of infestation (Inf), leaf rust (LR), effective fertile tiller (EFT), and single leaf area (SLA) had higher (> 20%) PCV estimates. In addition, PCV estimate is by far greater than the corresponding GCV values in all the traits considered. Similarly, estimate of genotype environment coefficients of variation (GECV) showed a slightly wide range of variations among the traits considered (Table 5).

### Estimates of heritability in broad sense and genetic advance

Estimate of heritability in broad sense (H^2^%) in the quantitative traits considered revealed a medium (44.82 in thousand seed weight or TSW) to lowe (38.51 in DTH and less in others) and a wide range of variation (0.00% in leaf width (LW) to 44.82% in thousand seed weight (TSW). Similarly, estimates of genetic advance (GA) revealed a wide range of variation (0.00 in leaf width LW to 26.62 in seed yield per hectare of land or SYPH). A similar trend of wide range has been shown in genetic advance as a per cent of traits mean (GA as % mean) (0.00 in leaf width or LW to 11.01 in thousand seed weight or TSW) (Table 5).

### Analysis of correlation coefficients

Result of the pairwise correlation coefficients between the quantitative traits studied is presented in Table 6. In this regard, considerable number of the traits showed significant (*P* < *0.05*) phenotypic (below diagonal) pairwise correlations. However, only few traits showed significant genotypic pairwise correlations. Seed yield per hectare of land (SYPH), one of the important quantitative traits, is among the traits that showed a significant (*P* < *0.05*) and positive phenotypic correlation with traits such as single leaf area (SLA) (0.06), thousand seed weight (TSW) (0.84), recovery rate per stand (RPS) (0.12), number of grains per plant (NGPP) (0.08) and a significant to highly significant negative correlation with days to emergence (DTE) (-0.05) (*P* < *0.05*), and rate of infestation (Inf) (-0.07) (*P* < *0.001*). Similarly, it showed a highly significant (*P* < *0.001*) and positive genotypic correlation with only two traits such as single leaf area (SLA) (0.21), and thousand seed weight (TSW) (0.84). Despite the significance level, the extents or magnitude of correlation sounds smaller (*r* < *0.5*) in all those traits and most of the remaining except SYPH vs TSW (*r* = 0.84), and RPS vs Inf (*r* = -0.81 for phenotypic and *r* = -0.80 for genotypic).

**Table 6:** Phenotypic (below diagonal) and genotypic (above diagonal) pairwise correlation coefficients for the traits using combined data

Variable	DTE	DTH	DTM	PH	LW	FLL	LN	SLA	NGPP	SL	AL	EFT	TSW	SYPH	NB	LR	Inf	RPS
DTE	**1.00**	0.12	0.13	-0.16	0.02	0.00	-0.11	-0.07	0.05	0.13	0.10	0.01	-0.14	-0.11	-0.02	-0.06	0.03	-0.10
DTH	0.03	**1.00**	0.11	-0.07	-0.05	0.03	0.06	0.00	-0.07	0.14	0.04	0.00	-0.03	-0.03	-0.11	0.03	0.14	-0.08
DTM	0.09***	-0.06***	**1.00**	-0.39***	0.09	-0.01	0.05	0.14	-0.01	0.02	-0.01	-0.28***	0.09	0.06	0.11	-0.08	0.13	-0.23***
PH	-0.30***	-0.12***	0.15***	**1.00**	0.02	0.12	-0.09	0.01	0.15	-0.05	0.00	0.15	0.06	0.04	-0.11	-0.06	-0.05	0.11
LW	-0.03	-0.04	0.02	0.02	**1.00**	0.13	-0.09	0.08	0.09	0.04	0.04	0.02	0.06	0.03	-0.14	0.13	0.01	-0.01
FLL	-0.02	0.14***	0.01	0.15***	0.02	**1.00**	-0.07	0.60***	0.16	0.09	0.06	-0.08	0.16	0.06	0.01	0.11	-0.08	0.13
LN	-0.04	0.35***	-0.05**	-0.07**	0.01	0.21***	**1.00**	-0.05	0.01	-0.02	0.05	-0.12	-0.17	-0.15	0.01	0.05	0.06	-0.03
SLA	-0.05**	0.07**	0.18***	0.15***	0.08***	0.65***	0.26***	**1.00**	0.05	-0.02	-0.09	-0.06	0.32***	0.21**	0.00	0.05	-0.03	0.06
NGPP	0.05**	0.04	0.03	-0.04	0.03	0.05**	0.02	0.01	**1.00**	-0.04	0.04	0.00	-0.01	-0.05	-0.10	-0.15	-0.11	0.15
SL	0.04	0.24***	-0.05	0.06**	-0.01	0.21***	0.16***	0.13***	0.01	**1.00**	0.19**	-0.01	0.02	0.03	0.04	0.14	0.00	0.05
AL	0.01	0.17***	-0.16***	0.01	-0.04	0.19***	0.14***	0.07**	-0.02	0.12***	**1.00**	0.03	-0.06	-0.07	0.04	0.05	-0.05	0.08
EFT	-0.01	0.25***	-0.16***	0.07**	0.00	0.21***	0.18***	0.14***	0.03	0.26***	0.17***	**1.00**	-0.05	0.06	-0.11	-0.11	-0.03	0.11
TSW	-0.05**	-0.01	0.02	-0.02	0.03	0.02	-0.01	0.04	0.08**	-0.02	-0.03	-0.04	**1.00**	0.84***	0.03	0.07	0.01	0.08
SYPH	-0.05**	-0.02	0.01	-0.02	0.03	0.03	-0.01	0.06**	0.08***	0.00	-0.01	0.00	0.84***	**1.00**	0.03	0.03	0.00	0.03
NB	-0.01	0.05	-0.04	-0.04	-0.02	-0.05	0.03	-0.06**	-0.04	0.09**	0.05**	-0.01	0.06**	0.04	**1.00**	0.09	0.07	-0.09
LR	0.03	0.02	0.00	-0.02	-0.02	-0.02	-0.01	-0.02	-0.03	0.06**	0.02	-0.03	0.00	-0.04	0.04	**1.00**	-0.06	0.11
Inf	0.08**	0.09***	0.02	-0.01	-0.01	0.02	0.01	0.00	-0.03	0.00	0.00	-0.03	-0.01	-0.07***	0.05**	0.04	**1.00**	-0.80***
RPS	-0.06**	-0.05**	-0.04	0.02	0.03	0.02	0.00	0.03	0.06**	0.02	0.02	0.04	0.11***	0.12***	-0.04	-0.02	-0.81***	**1.00**

^*^significant at *p* < 0.05

^**^highly significant at *p* < 0.01

^***^highly significant at *p* < 0.001

^*^Description of the traits is presented under Table 3

### Principal Components Analysis (PCA)

PC analysis, conducted using the 18 standardized quantitative traits revealed that the first eight principal axes (eigenvalue ≥ 1.06**)** accounted for 69% of the total variation (Table 7). The first principal component (PC1) accounted for 14.00% of the total variation and had high contributing factor loadings from thousand seed weight (TSW) (0.50), single leaf area (SLA) (0.48), and seed yield per hectare (SYPH) (0.45). The second PC axis accounted for 12.00% of the total variation and differentiated the accessions largely on the bases of recovery rate per stand (RPS) (0.52), rate of infestation (Inf) (-0.49), and days to maturity (DTM) (-0.42). The third PC axis contributed 9.00% of the total variation and had greater contributing factor loadings from seed yield per hectare (SYPH) (0.34), and flag leaf length (FLL) (-0.31). The fourth and fifth PC axes each accounted for 8.00% and 7.00% (in that order) of the total variation and differentiated the accessions largely on the bases of leaf rust (LR) (-0.41), number of grains per plant (NGPP) (0.35), net blotch (NB) (-0.33), spike length (SL) (-0.44). The sixth, seventh and eighth pcs axes accounted for 7.00%, 6.00% and 6.00%, respectively, of the total variation and had greater contributing factors from flag leaf length (FLL) (0.42) (sixth), leaf rust (LR) (0.36) (seventh), and leaf number (LN) (-0.54), days to heading (DTH) (-0.44) (eighth) (Table 7).

**Table 7:** PC analysis showing estimates of contribution from each trait to the principal components, and extents of variation on the first eight principal components

Variable^a^	PC1	PC2	PC3	PC4	PC5	PC6	PC7	PC8
DTE	-0.11	-0.12	-0.26	0.06	-0.35	-0.36	-0.19	0.22
DTH	-0.05	-0.14	-0.15	-0.03	-0.38	-0.06	0.33	-0.44
DTM	0.07	-0.42	-0.24	0.01	0.16	-0.24	-0.23	-0.15
PH	0.04	0.33	0.29	0.16	-0.23	0.36	-0.06	-0.04
LW	0.34	-0.18	-0.13	0.27	0.07	-0.23	0.31	0.13
FLL	0.33	0.07	-0.31	0.20	-0.18	0.42	-0.10	0.09
LN	-0.16	-0.07	-0.15	-0.04	0.26	0.29	0.13	-0.54
SLA	0.48	-0.09	-0.23	0.30	0.01	0.19	0.11	0.12
NGPP	0.04	0.18	-0.11	0.35	-0.08	0.02	-0.50	-0.27
SL	0.02	-0.02	-0.28	-0.31	-0.44	0.03	0.05	0.02
AL	-0.09	0.08	-0.25	-0.25	-0.33	0.08	-0.26	-0.09
EFT	-0.04	0.23	0.24	0.10	-0.32	-0.19	0.25	0.20
TSW	0.50	-0.06	0.27	-0.27	-0.06	-0.08	-0.15	-0.20
SYPH	0.45	-0.05	0.34	-0.31	-0.10	-0.15	-0.13	-0.19
NB	-0.01	-0.14	-0.04	-0.33	0.18	0.25	-0.32	0.42
LR	0.09	0.02	-0.20	-0.41	0.06	0.26	0.36	0.14
Inf	-0.10	-0.49	0.28	0.12	-0.26	0.29	0.02	0.02
RPS	0.15	0.52	-0.25	-0.13	0.18	-0.22	0.05	-0.08
Eigenvalue	2.46	2.18	1.61	1.45	1.34	1.26	1.15	1.06
Proportion	0.14	0.12	0.09	0.08	0.07	0.07	0.06	0.06
Cumulative	0.14	0.26	0.35	0.43	0.50	0.57	0.63	0.69

^a^Description of the variables is given under Table 3

**Fig. 1:**
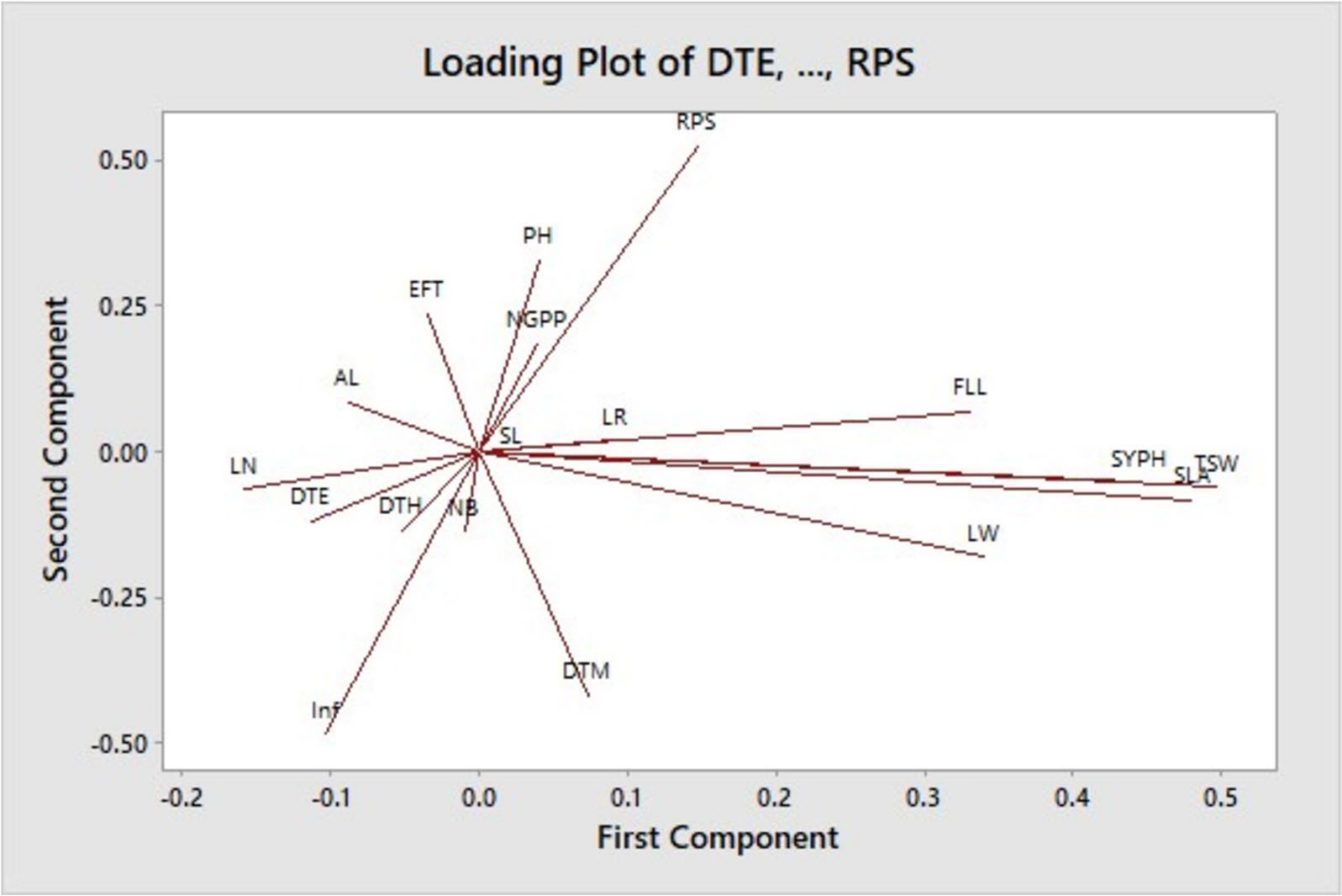
PCA loading plot showing the association between or among the traits (abbreviated letters) considered; Description of the traits is given under Table 3

PCA loading plot showed a loose positive and negative correlation among the traits considered (Fig. 1). For example, single leaf area (SLA), leaf width (LW), flag leaf length (FLL) and seed yield per hectare (SYPH) had a weak positive association with each other. Similarly, rate of infestation (Inf) and recovery per stand (RPS) showed a strong negative association as did effective fertile tiller (EFT) and days to maturity (DTM)*.* On the other hand, three traits such as seed yield per hectare (SYPH), thousand seed weight (TSW), and single leaf area (SLA) showed a strong and positive association.

**Fig. 2:**
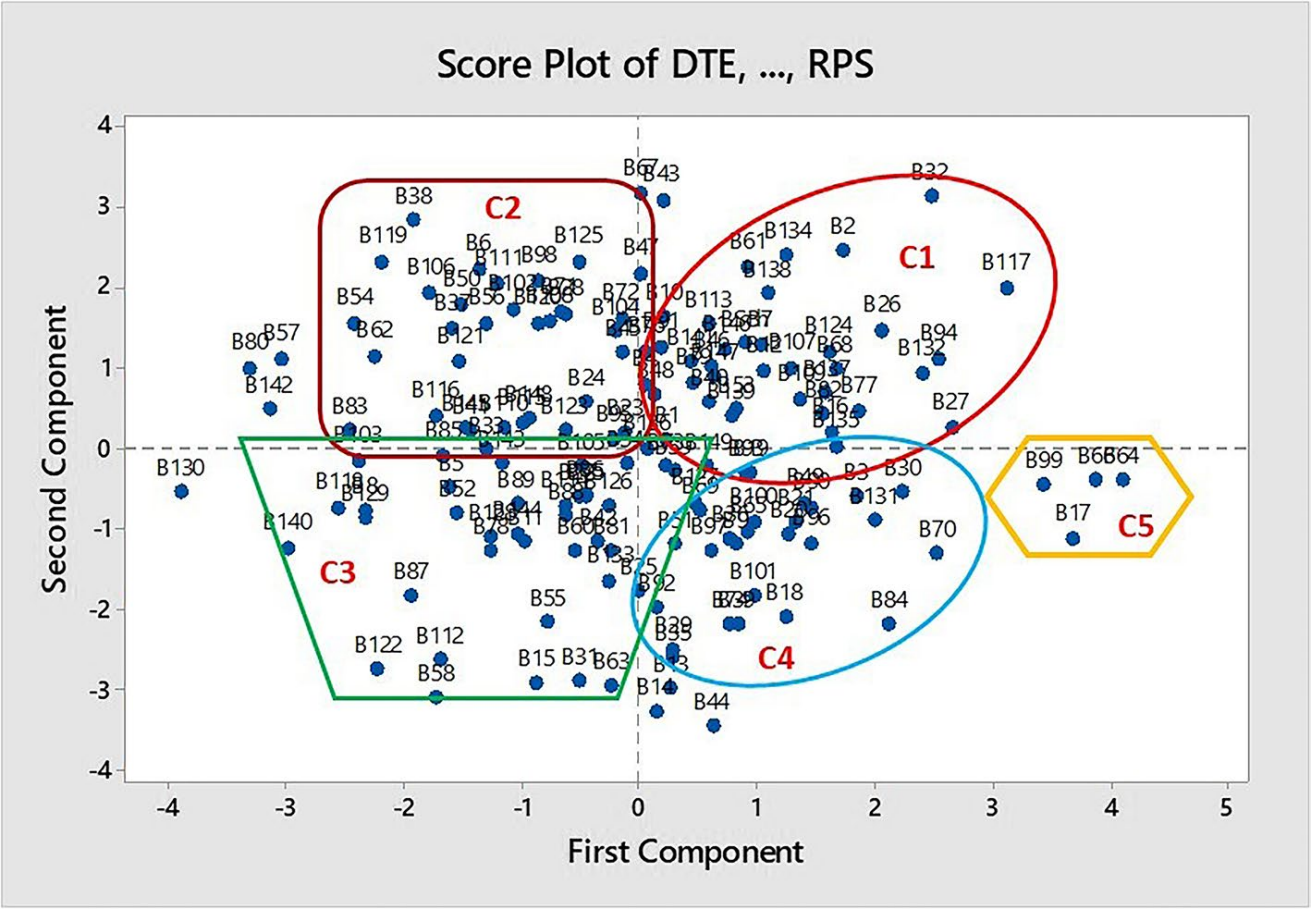
PCA Score plot showing the clustering pattern of the accessions (black dots with numbers) considered; Details of the accession codes is presented under Table 3

**Fig. 3:**
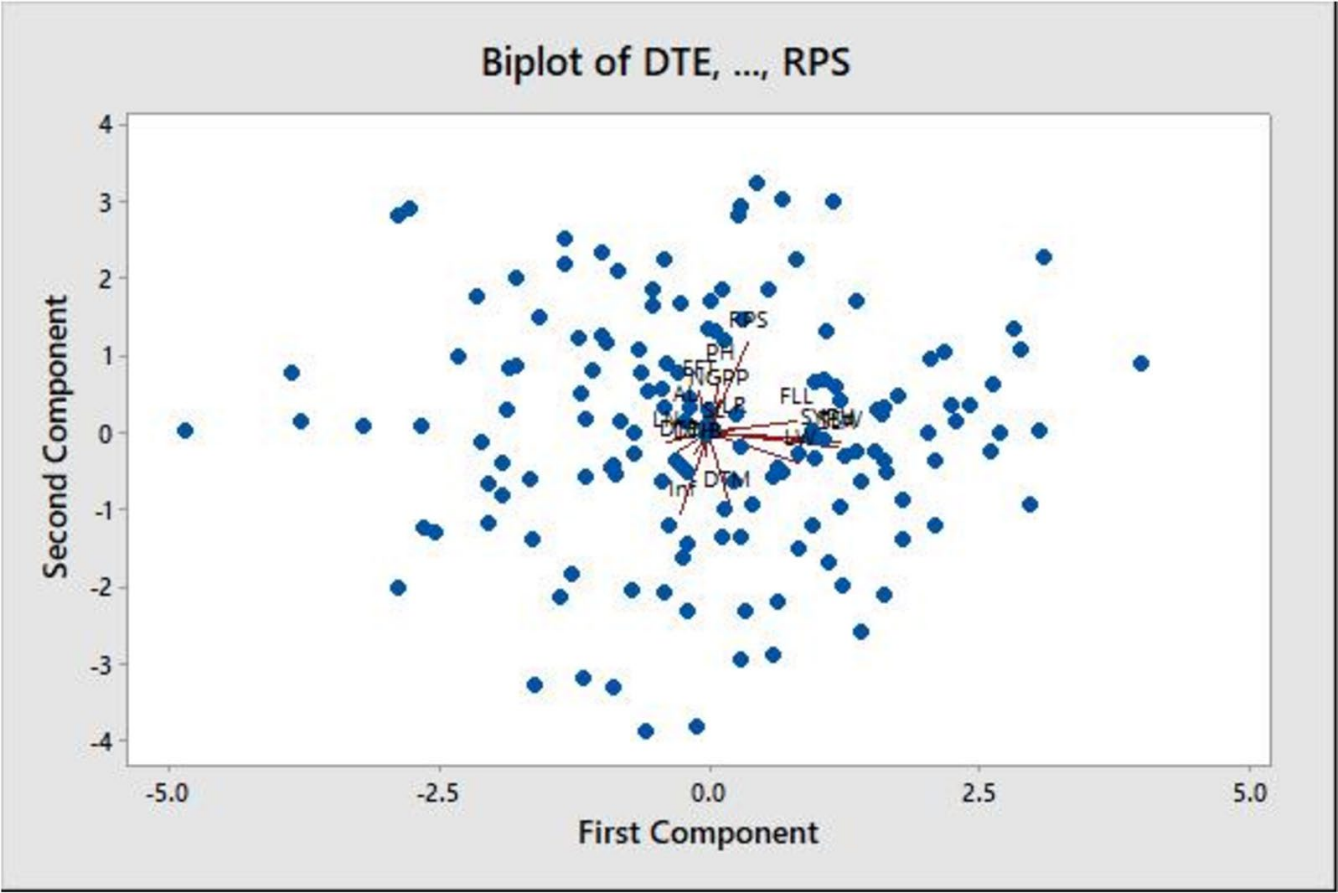
PCA biplot showing the pattern of association between PC scores of samples or the accessions used (black dots) and loadings of variables or the quantitative traits considered (abbreviated letters); Details of the accessions (black dots) and traits (abbreviated letters) is presented under Tables 1 and 3, respectively

PCA score plot grouped the accessions nearly into five clusters (Fig. 2). The grouping pattern was not parallel to the specific locations of collections. PCA biplot also revealed a weak association or contribution of most of the traits to the grouping patterns of the accessions (Fig. 3).

### Cluster analysis

**Fig. 4:**
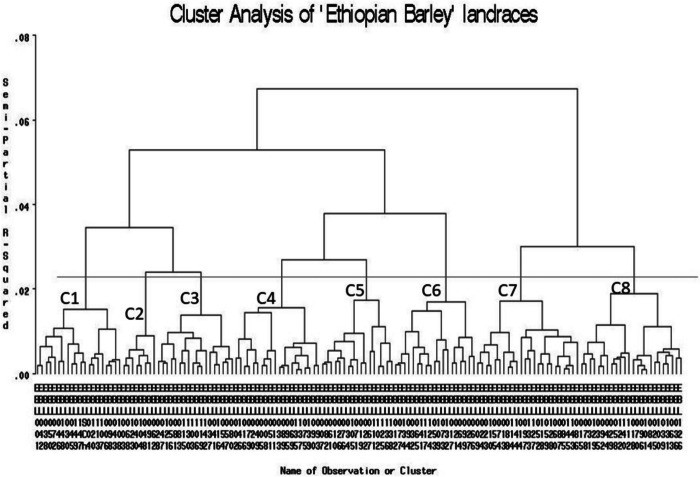
Cluster analysis of the 150 barley accessions considered; List of accessions on each cluster is presented below under Table 8

Cluster analysis was conducted using standardized data in order to have a good picture of the genetic association between the accessions studied. Accordingly, the patterns of grouping of individual accessions revealed eight clusters in which larger number of accessions were grouped under clusters 1 and 3 (C1 and C3; each contained 24 accessions or 16% of the total). Clusters 2 and 7 (C2 and C7) were the second largest (each contained 20 accessions or 13.33%), followed by clusters 8 (C8) (19), 5 (C5) (18) and 4 (C4) (17). The pattern seems poor in revealing specific geographic regions of origin (collection) of the accessions and hence, accessions from different specific localities appeared on the same cluster and vice versa (Fig. 4; Table 8).

**Table 8:** List of accessions included under each cluster

C1	C2	C3	C4	C5	C6	C7	C8
EBL096	EBL135	EBL085	EBL034	EBL106	EBL003	EBL001	EBL117
EBL101	EBL137	EBL145	EBL105	EBL119	EBL068	EBL042	EBL124
EBL035	EBL075	EBL088	EBL102	EBL041	EBL040	EBL006	EBL012
EBL092	EBL139	EBL114	EBL125	EBL116	EBL104	EBL098	EBL077
EBL070	EBL033	EBL060	EBL007	EBL047	EBL048	EBL004	EBL065
EBL039	EBL089	EBL087	EBL082	EBL121	EBL091	EBL120	EBL141
EBL131	EBL016	EBL143	EBL019	EBL103	EBL123	EBL045	EBL134
EBL029	EBL079	EBL023	EBL022	EBL083	EBL062	EBL149	EBL017
EBL058	EBL090	EBL110	EBL061	EBL115	-	EBL038	EBL021
EBL015	EBL093	EBL059	EBL136	EBL054	-	EBL050	EBL064
EBL078	EBL095	EBL128	EBL138	EBL057	-	EBL147	EBL099
EBL025	EBL005	EBL052	EBL010	EBL028	-	EBL150	EBL053
EBL100	EBL008	EBL118	EBL026	EBL056	-	EBL043	EBL109
EBL018	EBL020	EBL009	EBL071	EBL081	-	EBL046	EBL027
EBL031	EBL049	EBL024	EBL076	EBL080	-	EBL148	EBL066
EBL129	EBL069	EBL144	EBL067	EBL037	-	EBL108	EBL094
EBL122	EBL051	EBL055	EBL111	EBL142	-	EBL113	EBL073
EBL036	EBL002	EBL074	-	EBL130	-	EBL072	EBL132
EBL126	EBL146	EBL097	-	-	-	EBL107	EBL032
EBL140	EBL011	EBL133	-	-	-	EBL030	-
EBL044	-	EBL013	-	-	-	-	-
EBL063	-	EBL127	-	-	-	-	-
EBL084	-	EBL086	-	-	-	-	-
EBL112	-	EBL014	-	-	-	-	-

C stands for cluster; numbers associated indicate clusters 1 to 8

The estimate of pairwise generalized square distance between the clusters revealed moderate value and the range is nearly the same in several of the clusters. For example, clusters 1 and 3 (24.01, the largest distance), 6 and 7 (23.21), 5 and 8 (22.67), 3 and 6 (22.62), and 2 and 5 (21.83) revealed nearly closer pairwise distances. Clusters 1 and 3 showed the smallest pairwise distance (8.61). Similarly, intra (within) cluster distance among the accessions in each cluster showed a similar pattern (3.58 in cluster 3 to 4.24 in clusters 4 and 5) except for those accessions on cluster 6 that showed a relatively greater within cluster distance (6.13). Regarding estimate of mean distance, all the clusters are nearly equidistant from each other (14.17 in cluster 3 to 16.87 in cluster 1) except cluster 6 which is a bit distant from the others (mean cluster distance of 20.30) (Table 9).

**Table 9:** Estimate of pairwise inter, intra (diagonal element in bold) and mean cluster distance

**Cls**^a^	**1**	**2**	**3**	**4**	**5**	**6**	**7**	**8**	Mean
1	**3.67**								16.87
2	13.30	**4.13**							14.54
3	8.61	8.15	**3.58**						14.17
4	24.01^*^	12.31	16.35	**4.24**					16.38
5	19.37	21.83^*^	18.01	13.3	**4.24**				18.16
6	19.91	20.09	22.62^*^	19.69	16.51	6.13^**^			20.30
7	17.63	12.13	11.57	16.66	15.44	23.21^**^	**4.03**		15.65
8	15.22	13.98	13.87	12.33	22.67^*^	20.03^*^	12.91	**4.13**	15.86

^a^*Cls* clusters, * highly significant at P<0.05,  ** highly significant at P<0.01

## Discussions

### Germination efficiency of the *ex-situ* conserved landraces

While there is no clear distinction between specific storage conditions and the termination of life processes in different species, the storage conditions of different seeds have a significant impact on the termination of their life processes. In this regard [22], reported that there is no clearly specified duration for seed death because it is a gradual and cumulative process in which more and more cells die until certain critical parts of the seed become unable to perform their essential function. However, there is a general understanding regarding significant disparities in storage duration between orthodox and recalcitrant seeds where the first could maintain its moisture content for an extended period and remain viable as compared to the second type,which can only persist for a relatively shorter spanunless special storage practices are considered. Moore [23] reported that seed becomes less vigorous as the never-ceasing aging process moves onward toward death; but even long before death, the seed becomes questionable or worthless for planting purpose, especially under field conditions that are not highly favorable for germination and seedling development. In this regard, all the *ex-situ* conserved barley landraces considered exhibited a promising performance and revealed a good germination rate regardless of their long storage duration (36 to 57 years) at the EBI cold-room or longer years of collections (1964—1985) from the field. The result suggests good handling from the EBI which could be further enhanced as it is the only source for some germplasms which are totally missing from the farmer’s field. On the other hand, the present result is partly contradicting the general report by [24] suggesting the negative effects of long storage duration on germination rate and related issues and this could be partly attributed to the different environmental and genetic factors including storage temperature, seed moisture content, and genetic variability. The slight variation among the tested landraces seems normal since seed longevity vary among different genotypes, cultivars, and accessions because of genetic variations [25].

### Patterns of genetic variability in the landraces

The landraces studied showed a different pattern of genetic variability with respect to most of the qualitative morphological traits assessed, indicating the significantly stronger effects of selection pressure for various end-use qualities. For example, dominance of six-rowed types in the evaluated landraces partly suggests artificial selection pressure by farmers with the intention of obtaining more yield per plot of land. Similarly, larger number of the accessions with being long kernels and yellow or white color indicate preference of the farmers for different end-use qualities. Dominance of the accessions with erect growth habit indicates preference by the farmers because of their suitability for traditional and/or mechanized harvesting. Similarly, dominance of the accessions with white seed and glumes colors, as compared to the reddish and black types, once again qualify preference by the local community for different end use qualities. There are similar reports regarding the existence of wide variability in terms of frequency of those qualitative traits in Ethiopian barley accessions [13, 26, 27].

In general, the patterns of genetic variability in qualitative morphological traits suggest that the Ethiopian barley landraces, although conserved *ex situ* over a longer period of time, have higher morphological diversity, which is due to the country’s being the center of origin and having high ecological heterogeneity. This higher genetic variation and the good performance of ex-situ conserved landraces are key for selection breeding.

Together with qualitative traits, standard quantitative traits are among the important phenotypic markers that are widely used in breeding and conservation of plants, animals and other organisms. In this regard, most of the quantitative traits used to evaluate the *ex-situ* conserved Ethiopian barley landraces revealed a wide range of variability and wide ranges between the maximum and minimum mean values suggesting the ample variability in the landraces for further breeding work. Moreover, quantitative traits such as seed yield per hectare of land (SYPH), thousand seed weight (TSW), number of grains per plant (NGPP), number of effective fertile tillers (EFT) and disease related traits such as rate of infestation (Inf) and recovery per stand (RPS) are important targets of selection. In addition, analysis of variance (ANOVA) supported importance of those traits and others in targeted selective breeding as they showed a significant variation among the tested accessions. Similar results have been reported on the significant variations of several of the quantitative traits in Ethiopian barley landraces [28–31].

However, the existing variation could not be fully exploited as intended because of several genetic and environment related factors which are largely explained in terms of interactions between or among genes and interaction between genes and environmental factors. Consequently, the highly significant variation in mean square values for most of the traits considered over test years, locations, and year-location interactions observed along with interactions between accessions and test years, and locations signals the necessity of exercising the utmost caution and emphasizes the significance of testing the landraces at multiple locations over a couple of years to clearly indicate the amount of genetic based available variations for further use. There had been similar reports by [7, 13, 21, 32, 33] on different barley landraces from Ethiopia.

Similarly, the estimation of variance components is used to disclose the extent of genetic variation and the effects of interaction, especially interaction with the environment, for further uses. In this regard, the tested landraces showed wide range of variation in both phenotypic (δ^2^p) and genotypic (δ^2^g) variance estimates which is in agreement with results reported by [13, 34] on Ethiopian barley landraces collected from farmer’s field. Such significant and wide range of variations show the existence of large variability among the tested *ex-situ* conserved barley landraces in particular and barley landraces in Bale and Arsi zones in general. The significant and wide range of variations due to genotype-year (σ^2^gy), genotype-location (σ^2^gl), genotype-year-location (σ^2^gyl) interactions and error variance (σ^2^e) in some of the traits especially in seed yield per hectare of land (SYPH) revealed detectable impact of environmental factors to the variations. Thus, care should be taken while selecting the landraces for yield improvement though it has polygenic inheritance pattern. The high coefficient of genetic determination (R^2^) detected, particularly, in most of the yield contributing traits, also suggest possibility of identifying superior accessions with respect to the traits.

The insightful effect of environmental factors on the detected variation was further explicated by estimates of genotype-environment interaction (δ^2^gl) which was greater than zero and PCV values which were by far greater than the corresponding GCV values in all the traits. Such huge environment effect could be attributed to the current un predictable climate change which is quite different from the years back conditions when the landraces were collected. As a result, the *ex-situ* conserved landraces require some sort of multiple tastings to either develop adaptive potential though they are expected to have excelled adaptive and unique genes as compared to germplasms under cultivation.

Four traits such as rate of infestation (Inf), leaf rust (LR), effective fertile tiller (EFT), and single leaf area (SLA) had scored higher (> 20%) PCV and lower GCV estimate following [35]. This observation is in agreement with the result of [13, 36]. Similarly, the higher difference between the GCV and PCV estimates in these traits suggest the highly pronounced environmental influence. However, this finding is contradicting the reports of [13, 34] on barley landraces from farmers’ fields and thus, there might be differing environmental conditions from the situation when the landraces had been collected.

### Traits heritability and seed yield improvement

The concept of heritability pertains to the proportion of phenotypic variability that can be ascribed to genetic variability.. Its estimation is helpful in predicting the expected progress to be achieved through selection process since it indicates the heritable portion of the total variations which is the point of interest in morphological traits-based genetic performance analysis.

Its value could be very high (≥ 80%), moderately high (60–79%), medium (40–59%), or low (< 40%) following [37] benchmark. In this sense, all the traits considered except thousand seed weight (TSW) with a medium heritability, had low broad sense heritability (H^2^) estimates and eventually low GCV. The result implies that selection for the characters require special attention due to high environmental effects. Moreover, most of the traits had a direct link with seed yield per hectare of land (SYPH), an important trait for breeding, though it follows polygenic inheritance and thus, environmental factors and other quantitative traits should be seriously managed under the current unpredictable environmental conditions. Similar result has been reported by [13] on barley landraces from collected from farmers field in Ethiopia stressing the profound effect of environmental condition on traits’ heritability.

Genetic advance (GA) can be delineated as the enhancement of traits genotypic values for the new population that result from selection relative to the base population, under one cycle of selection at a given selection intensity [37]. To this end, estimates of GA for seed yield per hectare of land (SYPH) was 8.43 with the associated expected genetic advance values expressed as a percentage of the genotypes mean (GAM) of 0.37 which is low. GAM signposts the gain that could be expected from selection of the top 5% of the populations. Johnson et al. [38] categorized GAM as low (< 10%), moderate (10–20%), and high (> 20%). In this regard, all of the traits considered including seed yield per hectare of land (SYPH***)*** an important target trait for improvement had low estimate.

### Performance of the landraces in terms of pairwise correlation coefficients

The pairwise correlation coefficient analysis determines the extent and degree of the relationship between two characteristics. The association could be attributed to genotypic component, linkage between genes or gene effect [39], or to environmental effects (phenotypic), or both [40]. When determining how strongly traits are correlated, both correlation coefficients are crucial in determining whether selection for a given trait leads to either progress or retrogression, especially when it comes to quantitative traits like yield [41]. In this case, substantial number of the traits considered showed significant phenotypic and genotypic correlations. For example, seed yield per hectare of land (SYPH) is among those traits that showed significant positive and negative phenotypic and genotypic correlations with several traits. Those correlated traits, depending up on the magnitude of correlation, are important in improving the yield of barley landraces.

### Patterns of genetic relationship in the landraces

The extent and trends of genetic relatedness in any population or target sample can be revealed using cluster analysis methods, including PCA and cluster analysis. The practical application of PCA lies in its ability to identify the traits that have most contributed to the observed variation within a group of samples or populations. This makes it useful for selecting parental lines during breeding. With regards to its magnitude, traits with coefficients of the eigenvector close to one show a strong influence on a given trait and vice versa [42]. Therefore, traits with higher coefficients, typically 0.6 and above, on the PC axes should be considered more important [43]. Similarly, characters with higher factor loadings contribute more to the lumping together or scattering apart of accessions and thus, are given much attention on choosing the clusters for any desired purpose of breeding or conservation [44]. With this fact, the first eight principal axes (eigen value ≥ 1.06**)** accounted for 69.00% of the total variation. However, most of the traits in these PC axes had lower factor loadings (< 0.50) except four traits such as leaf number (LN), recovery per stand (RPN), thousand seed weight (TSW) and number of grains per plant (NGPP). These three traits are supposed to play a great role for the divergence and exhibited great influence on the phenotype of the accessions and could be targets of selection breeding. The result is concordant with the previous reports by [9, 25] using barley landrace collections from Ethiopia.

Likewise, cluster analysis is also used to show genetic relatedness between or among the subjects of study. In this regard, the *ex-situ* conserved barley landraces considered formed eight major genetic clusters with a weak trend of association between or among accessions from the same geographic location of collection and vice versa. Falconer [45] reported that variation in origin (geographical separation), ancestral relationship, gene frequency and morphology are the probable sources of genetic diversity. However, it is evidenced that, though genetic diversity is associated with geographical diversity, they are not necessarily directly related. To this end, the weak tendency of association between geographical proximity and genetic diversity of the accessions revealed a moderate divergence among the tested accessions.

## Conclusions

In recent days, Ethiopian barley landraces are diminishing at faster rate owing to environmental constraints and research focus of the country. Larger number of the landraces is found* ex-situ* conserved (one of the effective ways of preserving germplasms for longer duration) at the Ethiopian Biodiversity Institute (EBI), Ethiopia. In the present study, the landraces showed a promising germination rate and percentage regardless of their very long storage duration at the Institute. Moreover, the study generated basic information on the extents of their genetic variability that promotes the potentiality and high economic values of *ex situ* conserved barley landraces and promoted detailed studies at more locations over a couple of years to clearly exploit the actual genetic based variability in the current scenario of climate change. In addition, more robust molecular markers are mandatory to clearly reveal the genetic based variability for further utilizations.

## Materials and methods

### Experimental materials

The study involved a total of 150 *ex situ* conserved cultivated barley landrace accessions. The samples were obtained from the Ethiopian Biodiversity Institute (EBI), Addis Ababa, Ethiopia. The accessions had been collected from the Arsi-Bale highlands before 1986 and were assigned full passport data (Additional file 1)*.*

### Seed viability test

Seed germination test was conducted at the Biology Department Laboratory of Madda Walabu University (Bale-Robe, Ethiopia). Ten clean barley seeds of each accession were soaked in 75 mL distilled water in a separate sterile petri dish for 24 h. The seeds were then allowed to germinate on a Whatman filter paper at 20 °C for 7 days, following the procedures specified by the International Seed Testing Agency (ISTA) [46]. The experiment was conducted using a complete randomized design (CRD) in two replications.

### Field experiment

The field experiment was conducted under rain-fed conditions over two years (2021 and 2022) at three locations, namely Madda Walabu University (MWU) Integrated Research Field (Bale Robe, Ethiopia), Sinana Agricultural Research Institute (SARI) (Bale, Ethiopia), and Agarfa Agricultural Technical, Vocational and Education Training (TVET) College (Agarfa, Ethiopia). Description of those areas is presented below (Table 10).

**Table 10:** Detailed description of the experimental sites

**Parameters**		**Test locations**		
		**SARI**^**a**^	**Agarfa TVET**	**MWU**
Distance from Addis Ababa		460 km	458	430 km
Altitude (m.a.s.l)		2400	2358	2494
Mean annual temperature (minimum / maximum)		9.5 °C / 21 °C	8.6° C / 22.4° C	9.4 °C / 25.2 °C
Average annual rainfall		1174 mm	836.70 mm	860 mm
Soil texture		Cambisols with minor Vertisols	Vertisol and clay	Cambisol with minor Vertisol
Global positioning	Latitude	07°06′12"N	6° 67′ 11''N	7°08′13''N
	Longitude	40°12′40" E	40° 43′35''E	39°59′40''E

Source: NMSA (National Meteorological Service Authority) 2020

^a^*SARI* Sinana Agricultural Research Institute

First season seed planting were done from 5—15 June 2021. Similarly, second round seed planting were done from 8—16 June 2022. Harvesting was done from November 10 – 17, 2021 for first year planting and from November 14 – 21 for second year planting.

### Experimental design and procedures

The experiment was set up in an incomplete block design called alpha lattice, with two replicates per site. Each accession was grown in four rows forming a plot 2 m long and 1.2 m wide. The distances between the blocks, plots, rows and plants were 1.5 m, 0.6 m, 0.4 m and 10 cm respectively. Sowing was done by hand at the correct depth (3–6 cm) in the moist soil to cover the seeds evenly and thus maintain moist conditions for vigorous and healthy germination and growth.

### Data collection and statistical analysis

Physiological seed qualities such as standard germination and seed vigor tests were conducted following the International Seed Testing Agency (ISTA) [46] and Maguire [47]. Accordingly, the germination percentage was given as:

$$\mathrm{Germination\ }(\mathrm{\%})=\frac{\mathrm{Total\ number\ of\ normal\ seedlings }}{\mathrm{Total\ number\ of\ seeds\ sown}}\times 100$$

Seed vigor was evaluated by using seed germination rate, which relies on the quantity of normal seedlings and average germination time. Hence, seed germination rate was calculated following [47] which is given as Seed germination rate = ∑n/∑D, where n is the number of seeds germinated on day D out of 100 seeds sown, and D is the number of days counted from the beginning of the test.

Mean germination time (MGT) was calculated following [48]. Accordingly, $\mathrm{MGT}= \sum_{\mathrm{i}=1}^{\mathrm{k}}{\mathrm{n}}_{\mathrm{i}}{\mathrm{t}}_{\mathrm{i}}/ \sum_{\mathrm{i}=1}^{\mathrm{k}}{\mathrm{n}}_{\mathrm{i}}$, where ${\mathrm{n}}_{\mathrm{i}}$ is the number of seeds germinated at the time i; ${\mathrm{t}}_{\mathrm{i}}$ is the time from the start of the experiment to the i^th^ observation, and k is the time of last germination.

Field performance data was collected for a total of 29 traits, including 14 quantitative and 11 qualitative as well as four disease-related traits adopted from the standard barley descriptors [49].

The frequency analysis for the qualitative traits was carried out using MINITAB® Release 19 [50] statistical software. Following Hartley’s F-max based error variance homogeneity test [51], analyses of variance (ANOVA) were calculated for each site and combined across sites for quantitative and disease traits using the GLM procedure of the SAS software based on the following statistical model:

$${\mathbf{Y}}_{\mathbf{i}\mathbf{j}\mathbf{k}\mathbf{l}}={\varvec{\upmu}}+{\mathbf{b}}_{\mathbf{i}}+{\mathbf{g}}_{\mathbf{j}}+{\mathbf{l}}_{\mathbf{k}}+{\mathbf{y}}_{\mathbf{l}}+{\mathbf{g}\mathbf{l}}_{\mathbf{j}\mathbf{k}}+{\mathbf{g}\mathbf{y}}_{\mathbf{j}\mathbf{l}}+{\mathbf{g}\mathbf{y}\mathbf{l}}_{\mathbf{j}\mathbf{k}\mathbf{l}}+{\mathbf{e}}_{\mathbf{i}\mathbf{j}\mathbf{k}\mathbf{l}}$$

Where, b = effect of block i, g = effect of genotype (accession) j, l = effect of location k, y = effect of year l, gl = effect of interaction of genotype j by location k, gy = the effect of interaction of genotype j by year l, gyl = effect of interaction of genotype j, by year l, and location k, and e = effect of interaction of genotype by block i, genotype j, location k, and year l.

Locations, years, accessions and their interactions were considered as random variables in the analysis according to [52]. The variance components assigned to the accessions and their interactions were calculated using the VARCOMP procedure of SAS.

The estimation of environmental, genotypic and phenotypic variance components and their coefficients of variation per site and combined across sites was computed based on the methods of [53, 54]. Accordingly,

**Phenotypic variance (**${\delta }_{p}^{2}$**) per location **$={\delta }_{g}^{2}+{\delta }_{e}^{2}$ where, ${\delta }_{p}^{2}$ = phenotypic variance; ${\delta }_{g}^{2}$ = genotypic variance and ${\delta }_{e}^{2}$ = environmental variance = error variance

**Genotypic variance (**${\updelta }_{\mathrm{g}}^{2}$**) per location **$=({\mathrm{MS}}_{\mathrm{g}}-{\mathrm{MS}}_{\mathrm{e}})/\mathrm{r}$ where, ${\mathrm{MS}}_{\mathrm{g}}$ = mean square of genotype; ${\mathrm{MS}}_{\mathrm{e}}$ is mean square of error and $\mathrm{r}$ is the number of replications

**Phenotypic Coefficient of Variation (**$\mathrm{PCV }(\mathrm{\%})$**) per location **$=(\sqrt{{\updelta }_{\mathrm{p}}^{2}}/\mathrm{m})\mathrm\times100$, where, PCV = phenotypic coefficient of variation; m = population mean for the trait considered

**Genotypic Coefficient of Variation (**$\mathrm{GCV }(\mathrm{\%})$**) per location** = $(\sqrt{{\updelta }_{\mathrm{g}}^{2}}/\mathrm{m})\mathrm{\times}100$ where, $\mathrm{GCV}$ = genotypic coefficient of variation

**Genotypic variance (**${\updelta }_{\mathrm{g}}^{2}$**) combined over location** = ${\updelta }_{\mathrm{g}}^{2}=({\mathrm{MS}}_{\mathrm{g}}-{\mathrm{MS}}_{\mathrm{gl}})/\mathrm{rl}$, where, ${\mathrm{MS}}_{\mathrm{g}}$ = mean square of genotype; ${\mathrm{MS}}_{\mathrm{gl}}$ is mean square due to genotype by environment interaction; $\mathrm{l}$ = number of locations; $\mathrm{r}$ = number of replications

**G x E interaction variance (**${\updelta }_{\mathrm{gl}}^{2}$**) combined over location = **$({\mathrm{MS}}_{\mathrm{gl}}-{\mathrm{MS}}_{\mathrm{e}})/\mathrm{r}$, where, ${\mathrm{MS}}_{\mathrm{gl}}$ = mean square due to genotype by environment interaction; ${\mathrm{MS}}_{\mathrm{e}}$ = combined error means square (${\updelta }_{\mathrm{e}}^{2}$)

**Phenotypic variance (**${\delta }_{p}^{2}$**) combined over location** = ${\updelta }_{\mathrm{g}}^{2}+({\updelta }_{\mathrm{gl}}^{2}/\mathrm{l})+({\updelta }_{\mathrm{e}}^{2}/\mathrm{rl})$,

**Phenotypic coefficient of variance (**$\mathrm{PCV }(\mathrm{\%})$) **combined over location** = (√δ^2^p/m) × 100, $\mathrm{PCV }(\mathrm{\%})=(\sqrt{{\updelta }_{\mathrm{p}}^{2}}/\mathrm{m})\mathrm\times100$ where, PCV = phenotypic coefficient of variation; ${\updelta }_{\mathrm{p}}^{2}$ = phenotypic variance and $\mathrm{m}$ = population mean for the trait considered

**Genotypic coefficient of variation (**$\mathrm{GCV }(\mathrm{\%})$**) combined over locations** = (√δ^2^ g/m) × 100, $\mathrm{GCV}=(\sqrt{{\updelta }_{\mathrm{g}}^{2}}/\mathrm{m})\mathrm\times100$ where, GCV = genotypic coefficient of variation; ${\updelta }_{\mathrm{g}}^{2}$ = genotypic variance; $m$ = population mean for the trait considered

**G x E interaction coefficient of variation (**$\mathrm{GECV}$**)** = $(\sqrt{{\updelta }_{\mathrm{gl}}^{2}}/\mathrm{m})\mathrm\times100$ where ${\updelta }_{\mathrm{gl}}^{2}$, = genotypic x environment variance; $\mathrm{m}$ = population mean for the trait considered

**Heritability in broad sense (H**^**2**^** or h**^**2**^**)** was estimated according to [54] as: ${\mathrm{H}}^{2}=({\updelta }_{\mathrm{g}}^{2}/{\updelta }_{\mathrm{p}}^{2})\mathrm\times100$ where, ${\updelta }_{\mathrm{p}}^{2}={\updelta }_{\mathrm{g}}^{2}+({\updelta }_{\mathrm{gl}}^{2}/\mathrm{l})+({\updelta }_{\mathrm{e}}^{2}/\mathrm{rl})$

**Expected genetic advance** under selection assuming the selection intensity at 5% was also computed following [54] as: $\mathrm{GA}=(\mathrm{K})({\updelta }_{\mathrm{p}})({\mathrm{H}}^{2})$ where $\mathrm{GA}$ = expected genetic advance; $\mathrm{K}$ = selection differential that varies depending up on the selection intensity and stands at 2.056 for selecting 5% of the genotypes. ${\updelta }_{\mathrm{p}}$ = phenotypic standard deviation and, ${\mathrm{H}}^{2}$ = heritability in broad sense

**Genetic advance as percent of mean** was obtained by the formula of [55] as; **GA (% of mean) = (GA/m) × 100,** where, GA = genetic advance; $m$ = population mean for the trait considered

Phenotypic and genotypic correlation coefficients between two traits were determined by using PROC CANDISC procedure of SAS software following the variance and covariance components [53, 55].

${\mathrm{r}}_{\mathrm{p}(\mathrm{xy})}={\mathrm{COV}}_{\mathrm{P}(\mathrm{x},\mathrm{y})}/\sqrt{({\updelta }_{\mathrm{px}}^{2})({\updelta }_{\mathrm{py}}^{2})}$, where ${\mathrm{COV}}_{\mathrm{P}(\mathrm{x},\mathrm{y})}$ = phenotypic covariance between traits X and Y, ${\mathrm{r}}_{\mathrm{p}(\mathrm{xy})}$ = phenotypic correlation coefficient between traits X and Y, ${\updelta }_{\mathrm{px}}^{2}$**=** phenotypic variance of trait X; ${\updelta }_{\mathrm{py}}^{2}$**=** phenotypic variance of trait Y.

${\mathrm{r}}_{\mathrm{g}(\mathrm{xy})}={\mathrm{COV}}_{\mathrm{g}(\mathrm{x},\mathrm{y})}/\sqrt{({\updelta }_{\mathrm{gx}}^{2})({\updelta }_{\mathrm{gy}}^{2})}$, where ${\mathrm{COV}}_{\mathrm{g}(\mathrm{x},\mathrm{y})}$ = genotypic covariance between traits X and Y, ${\mathrm{r}}_{\mathrm{g}(\mathrm{xy})}$ = genotypic correlation coefficient between traits X and Y, ${\updelta }_{\mathrm{gx}}^{2}$**=** genotypic variance of trait X; ${\delta }_{gy}^{2}$**=** genotypic variance of trait Y.

Phenotypic and genotypic correlation coefficients were tested for significance using the formula proposed by [53, 56], using the t-table with (g-2) degrees of freedom at 5% and 1% significance levels; g is the number of genotypes (treatments) used in the study.

${\mathrm{t}}_{\mathrm{p}}={\mathrm{r}}_{\mathrm{p}(\mathrm{xy})}/{\mathrm{SE}}_{\mathrm{p}(\mathrm{xy})}$ and ${\mathrm{t}}_{\mathrm{g}}={\mathrm{r}}_{\mathrm{g}(\mathrm{xy})}/{\mathrm{SE}}_{\mathrm{g}(\mathrm{xy})}$, respectively, where ${\mathrm{SE}}_{\mathrm{p}(\mathrm{xy})}$ and ${\mathrm{SE}}_{\mathrm{g}(\mathrm{xy})}$, represent standard error for phenotypic and genotypic correlation, and were computed as:

${\mathrm{SE}}_{\mathrm{p}(\mathrm{xy})}=\sqrt{{(1-{\mathrm{r}}_{\mathrm{p}\left(\mathrm{xy}\right)})}^{2}/(2{\mathrm{H}}_{\mathrm{x}}{\mathrm{H}}_{\mathrm{y}})}$, and ${\mathrm{SE}}_{\mathrm{g}(\mathrm{xy})}=\sqrt{{(1-{\mathrm{r}}_{\mathrm{g}\left(\mathrm{xy}\right)})}^{2}/(2{\mathrm{H}}_{\mathrm{x}}{\mathrm{H}}_{\mathrm{y}})}$, where, ${\mathrm{H}}_{\mathrm{x}}$ and ${\mathrm{H}}_{\mathrm{y}}$ are heritability estimate for traits $\mathrm{x}$ and $\mathrm{y}$.

Multivariate analysis that includes clustering and PCA was also performed using SAS and Minitab software. Using SAS software version 9.0, the pseudo-F and pseudo-t^2^ statistics were used to calculate the number of clusters. The generalized Mahalanobis D^2^ statistic was used to determine the genetic distance between clusters:

${\mathrm{D}}_{\mathrm{p}}^{2}={({\overline{\mathrm{X}} }_{\mathrm{i}}-{\overline{\mathrm{X}} }_{\mathrm{j}})}^{\mathrm{T}}{\mathrm{S}}^{-1}({\overline{\mathrm{X}} }_{\mathrm{i}}-{\overline{\mathrm{X}} }_{\mathrm{j}})$ where, ${\mathrm{D}}_{\mathrm{p}}^{2}$ = total generalized distance based on p characters, ${\overline{\mathrm{X}} }_{\mathrm{i}}$ and ${\overline{\mathrm{X}} }_{\mathrm{j}}$ are the p (sample) mean vectors of accessions i and j, respectively, the superscript T denotes matrix transpose, and S denotes the (bias-corrected) sample covariance matrix of the n observations in the observed sample which is given as;

$\mathrm{S}=(\left({\mathrm{n}}_{1}-1\right){\mathrm{S}}_{1}+\left({\mathrm{n}}_{2}-1\right){\mathrm{S}}_{2})/\mathrm{N}$, [57] where, ${\mathrm{n}}_{1}$ and ${\mathrm{n}}_{2}$ represent random samples of sizes drawn from groups G1 and G2, ${\mathrm{S}}_{1}$ and ${\mathrm{S}}_{2}$ designate (bias-corrected) sample covariance matrices, and $\mathrm{N}={\mathrm{n}}_{1}+{\mathrm{n}}_{2}-2$.

The D^2^ value for the cluster pairs was considered as a calculated chi-squared value (χ^2^) and tested for significance at the required probability level against the tabulated values of χ^2^ for p degrees of freedom (d.f = n-1), where p is the number of characteristics considered [53].

Principal component analysis (PCA) was performed for the combined and standardized accession mean using MINITAB® Release 19 statistical software [50].

### Supplementary Information

**Additional file 1. **List of the ex-situ conserved barley landraces, along with their passport data, considered in the present study.

## Data Availability

The list of accessions used during the current study is included as “Additional file 1”. Further requests for the raw data and other essential materials can be accessed from the corresponding author; Fekadu Gadissa, e-mail fikega2000@gmail.com, Phone- + 251 911 909582.

## Abbreviations

ANOVA: Analysis of variance
EBI: Ethiopian Biodiversity Institute
GCV: Genotypic Coefficient of variance
NMSA: National Meteorology Service Authority
PCA: Principal Components Analysis
PCV: Phenotypic Coefficient of variance
