# Supplementary material for: Phenotypic characterization and seed viability test in ex-situ conserved Ethiopian cultivated barley (Hordeum vulgare L.) landraces
Source: BMC Plant Biol. 2023 Dec 4;23:613. doi: 10.1186/s12870-023-04628-7 (PMC10694994; doi:10.1186/s12870-023-04628-7)
Supplement: Supplementary file 1 — Additional file 1. List of the ex-situ conserved barley landraces, along with their passport data, considered in the present study. [file 12870_2023_4628_MOESM1_ESM.docx]

**Supplementary file 1: List of the ex-situ conserved barley landraces, along with their passport data, considered in the present study**

| S/N | Acc. | Entry code | Year of collection | Country Code | Region | Zone | Woreda/District | Former administrative woreda/zone | Latitude | Longitude | Altitude  m.a.s.l |
| --- | --- | --- | --- | --- | --- | --- | --- | --- | --- | --- | --- |
| 1 | 1066 | EBL001 | 1964 | ETH | Oromia | BALE | GOBA | Bale | 07-05-00-N | 40-02-00-E | 2385 |
| 2 | 1113 | EBL002 | 1964 | ETH | Oromia | BALE | GOBA | Bale | 09-05-00-N | 40-02-00-E | 2375 |
| 3 | 1114 | EBL003 | 1964 | ETH | Oromia | BALE | GOBA | Bale | 09-05-00-N | 40-02-00-E | 2410 |
| 4 | 1115 | EBL004 | 1964 | ETH | Oromia | BALE | GOBA | Bale | 09-05-00-N | 40-02-00-E | 2410 |
| 5 | 1116 | EBL005 | 1964 | ETH | Oromia | BALE | GOBA | Bale | 09-05-00-N | 40-02-00-E | 2500 |
| 6 | 1117 | EBL006 | 1964 | ETH | Oromia | BALE | GOBA | Bale | 09-05-00-N | 40-02-00-E | 2500 |
| 7 | 1118 | EBL007 | 1964 | ETH | Oromia | BALE | GOBA | Bale | 09-05-00-N | 40-00-00-E | 1650 |
| 8 | 1119 | EBL008 | 1964 | ETH | Oromia | BALE | GOBA | Bale | 09-05-00-N | 40-00-00-E | 1630 |
| 9 | 1121 | EBL009 | 1964 | ETH | Oromia | BALE | GOBA | Bale | 09-05-00-N | 40-00-00-E | 2100 |
| 10 | 1122 | EBL010 | 1964 | ETH | Oromia | BALE | GOBA | Bale | 09-05-00-N | 40-00-00-E | 2260 |
| 11 | 1123 | EBL011 | 1964 | ETH | Oromia | BALE | GOBA | Bale | 09-05-00-N | 40-00-00-E | 2600 |
| 12 | 1124 | EBL012 | 1964 | ETH | Oromia | BALE | GOBA | Bale | 09-05-00-N | 40-00-00-E | 2600 |
| 13 | 1233 | EBL013 | 1964 | ETH | Oromia | W. Arsi | ADABA | Bale | 07-02-00-N | 38-33-00-E | 2500 |
| 14 | 1275 | EBL014 | 1964 | ETH | Oromia | W. Arsi | ADABA | Bale | 07-02-00-N | 38-33-00-E | 2500 |
| 15 | 1309 | EBL015 | 1964 | ETH | Oromia | BALE | GOBA | Bale | 07-05-00-N | 40-02-00-E | 2450 |
| 16 | 1467 | EBL016 | 1964 | ETH | Oromia | BALE | GOBA | Bale | 07-05-00-N | 40-00-00-E | 2450 |
| 17 | 1527 | EBL017 | 1964 | ETH | Oromia | BALE | GOBA | Bale | 07-05-00-N | 40-00-00-E | 2450 |
| 18 | 1555 | EBL018 | 1964 | ETH | Oromia | BALE | GOBA | Bale | 07-05-00-N | 40-02-00-E | 2470 |
| 19 | 1556 | EBL019 | 1964 | ETH | Oromia | BALE | GOBA | Bale | 07-05-00-N | 40-02-00-E | 2410 |
| 20 | 1557 | EBL020 | 1964 | ETH | Oromia | BALE | GOBA | Bale | 07-05-00-N | 40-00-00-E | 2410 |
| 21 | 1578 | EBL021 | 1964 | ETH | Oromia | BALE | RAYTU | Bale | 07-01-00-N | 39-15-00-E | 2460 |
| 22 | 1599 | EBL022 | 1964 | ETH | Oromia | BALE | RAYTU | Bale | 07-02-00-N | 39-14-00-E | 2856 |
| 23 | 1723 | EBL023 | 1978 | ETH | Oromia | BALE | RAYTU | Bale | 06-59-00-N | 39-10-00-E | 2470 |
| 24 | 3245 | EBL024 | 1979 | ETH | Oromia | W. Arsi | ADABA | Bale | 07-04-00-N | 39-38-00-E | 3370 |
| 25 | 3246 | EBL025 | 1979 | ETH | Oromia | W. Arsi | ADABA | Bale | 07-04-00-N | 39-38-00-E | 3410 |
| 26 | 3247 | EBL026 | 1979 | ETH | Oromia | W. Arsi | ADABA | Bale | 07-05-00-N | 39-39-00-E | 3460 |
| 27 | 3248 | EBL027 | 1979 | ETH | Oromia | BALE | SINANA | Bale | 07-02-00-N | 39-34-00-E | 3570 |
| 28 | 3249 | EBL028 | 1979 | ETH | Oromia | BALE | DINSHO | Bale | 07-05-00-N | 39-49-00-E | 3000 |
| 29 | 3250 | EBL029 | 1979 | ETH | Oromia | BALE | DINSHO | Bale | 07-05-00-N | 39-50-00-E | 2970 |
| 30 | 3251 | EBL030 | 1979 | ETH | Oromia | BALE | DINSHO | Bale | 07-05-00-N | 39-51-00-E | 2930 |
| 31 | 3252 | EBL031 | 1979 | ETH | Oromia | BALE | DINSHO | Bale | 07-05-00-N | 39-51-00-E | 2840 |
| 32 | 3253 | EBL032 | 1979 | ETH | Oromia | BALE | DINSHO | Bale | 07-07-00-N | 39-57-00-E | 2770 |
| 33 | 3254 | EBL033 | 1979 | ETH | Oromia | BALE | DINSHO | Bale | 07-07-00-N | 39-57-00-E | 2770 |
| 34 | 3255 | EBL034 | 1979 | ETH | Oromia | BALE | DINSHO | Bale | 07-09-00-N | 39-58-00-E | 2470 |
| 35 | 3256 | EBL035 | 1979 | ETH | Oromia | BALE | AGARFA | Bale | 07-16-00-N | 39-59-00-E | 2560 |
| 36 | 3257 | EBL036 | 1979 | ETH | Oromia | BALE | AGARFA | Bale | 07-20-00-N | 39-45-00-E | 2580 |
| 37 | 3258 | EBL037 | 1979 | ETH | Oromia | BALE | AGARFA | Bale | 07-17-00-N | 39-49-00-E | 2450 |
| 38 | 3259 | EBL038 | 1979 | ETH | Oromia | BALE | AGARFA | Bale | 07-17-00-N | 39-50-00-E | 2450 |
| 39 | 3260 | EBL039 | 1979 | ETH | Oromia | BALE | AGARFA | Bale | 07-22-00-N | 39-50-00-E | 2430 |
| 40 | 3261 | EBL040 | 1979 | ETH | Oromia | E. BALE | G. GOLELCHA | Bale | 07-22-00-N | 39-51-00-E | 2385 |
| 41 | 3262 | EBL041 | 1979 | ETH | Oromia | E. BALE | G. GOLELCHA | Bale | 07-19-00-N | 40-10-00-E | 2375 |
| 42 | 3263 | EBL042 | 1979 | ETH | Oromia | E. BALE | G. GOLELCHA | Bale | 07-18-00-N | 40-07-00-E | 2410 |
| 43 | 3264 | EBL043 | 1979 | ETH | Oromia | BALE | S. DINSHO | Bale | 07-10-00-N | 39-59-00-E | 2410 |
| 44 | 3265 | EBL044 | 1979 | ETH | Oromia | BALE | S. DINSHO | Bale | 07-04-00-N | 40-11-00-E | 2500 |
| 45 | 3266 | EBL045 | 1979 | ETH | Oromia | BALE | S. DINSHO | Bale | 07-04-00-N | 40-11-00-E | 2500 |
| 46 | 3267 | EBL046 | 1979 | ETH | Oromia | BALE | GINIR | Bale | 06-59-00-N | 40-31-00-E | 1650 |
| 47 | 3268 | EBL047 | 1979 | ETH | Oromia | BALE | GINIR | Bale | 07-09-00-N | 40-47-00-E | 1630 |
| 48 | 3269 | EBL048 | 1979 | ETH | Oromia | BALE | GINIR | Bale | 07-10-00-N | 40-39-00-E | 2100 |
| 49 | 3270 | EBL049 | 1979 | ETH | Oromia | BALE | GINIR | Bale | 07-15-00-N | 40-28-00-E | 2260 |
| 50 | 3271 | EBL050 | 1979 | ETH | Oromia | BALE | GINIR | Bale | 07-15-00-N | 40-29-00-E | 2115 |
| 51 | 3272 | EBL051 | 1979 | ETH | Oromia | BALE | GINIR | Bale | 07-19-00-N | 40-31-00-E | 2050 |
| 52 | 3273 | EBL052 | 1979 | ETH | Oromia | E. BALE | G. GOLELCHA | Bale | 07-19-00-N | 40-30-00-E | 2030 |
| 53 | 3274 | EBL053 | 1979 | ETH | Oromia | E. BALE | G. GOLELCHA | Bale | 07-19-00-N | 40-31-00-E | 2020 |
| 54 | 3275 | EBL054 | 1979 | ETH | Oromia | E. BALE | G. GOLELCHA | Bale | 07-26-00-N | 40-32-00-E | 2080 |
| 55 | 3276 | EBL055 | 1979 | ETH | Oromia | E. BALE | G. GOLELCHA | Bale | 07-27-00-N | 40-33-00-E | 2040 |
| 56 | 3277 | EBL056 | 1979 | ETH | Oromia | E. BALE | G. GOLELCHA | Bale | 07-40-00-N | 40-42-00-E | 2220 |
| 57 | 3278 | EBL057 | 1979 | ETH | Oromia | E. BALE | G. GOLELCHA | Bale | 07-40-00-N | 40-38-00-E | 2090 |
| 58 | 3279 | EBL058 | 1979 | ETH | Oromia | E. BALE | G. GOLELCHA | Bale | 07-32-00-N | 40-40-00-E | 1985 |
| 59 | 3280 | EBL059 | 1979 | ETH | Oromia | E. BALE | G. GOLELCHA | Bale | 07-29-00-N | 40-29-00-E | 2100 |
| 60 | 3281 | EBL060 | 1979 | ETH | Oromia | BALE | GINIR | Bale | 07-23-00-N | 40-26-00-E | 2300 |
| 61 | 3282 | EBL061 | 1979 | ETH | Oromia | BALE | GINIR | Bale | 07-25-00-N | 40-23-00-E | 2360 |
| 62 | 3283 | EBL062 | 1979 | ETH | Oromia | E. BALE | G. GOLELCHA | Bale | 07-25-00-N | 40-24-00-E | 2410 |
| 63 | 3284 | EBL063 | 1979 | ETH | Oromia | BALE | GINIR | Bale | 07-22-00-N | 40-14-00-E | 2480 |
| 64 | 3285 | EBL064 | 1979 | ETH | Oromia | BALE | GINIR | Bale | 07-22-00-N | 40-14-00-E | 2480 |
| 65 | 3286 | EBL065 | 1979 | ETH | Oromia | BALE | GOBA | Bale | 07-01-00-N | 39-56-00-E | 2750 |
| 66 | 3287 | EBL066 | 1979 | ETH | Oromia | BALE | GOBA | Bale | 07-02-00-N | 39-56-00-E | 2750 |
| 67 | 3288 | EBL067 | 1979 | ETH | Oromia | BALE | GOBA | Bale | 07-00-00-N | 39-53-00-E | 2820 |
| 68 | 3289 | EBL068 | 1979 | ETH | Oromia | BALE | GOBA | Bale | 06-57-00-N | 39-59-00-E | 2840 |
| 69 | 3290 | EBL069 | 1979 | ETH | Oromia | BALE | ADABA | Bale | 06-59-00-N | 39-23-00-E | 2470 |
| 70 | 3291 | EBL070 | 1979 | ETH | Oromia | BALE | ADABA | Bale | 06-58-00-N | 06-58-00-E | 2630 |
| 71 | 3292 | EBL071 | 1979 | ETH | Oromia | BALE | ADABA | Bale | 06-58-00-N | 39-24-00-E | 2630 |
| 72 | 3293 | EBL072 | 1979 | ETH | Oromia | BALE | ADABA | Bale | 06-55-00-N | 39-29-00-E | 2830 |
| 73 | 3294 | EBL073 | 1979 | ETH | Oromia | BALE | ADABA | Bale | 06-55-00-N | 39-29-00-E | 2830 |
| 74 | 3295 | EBL074 | 1979 | ETH | Oromia | BALE | ADABA | Bale | 06-55-00-N | 39-29-00-E | 2830 |
| 75 | 3296 | EBL075 | 1979 | ETH | Oromia | BALE | ADABA | Bale | 07-03-00-N | 39-24-00-E | 2450 |
| 76 | 3642 | EBL076 | 1979 | ETH | Oromia | BALE | AGARFA | Bale | 07-16-00-N | 39-49-00-E | 2633 |
| 77 | 3643 | EBL077 | 1979 | ETH | Oromia | BALE | AGARFA | Bale | 07-16-00-N | 39-49-00-E | 2745 |
| 78 | 3646 | EBL078 | 1979 | ETH | Oromia | BALE | S. DINSHO | Bale | 07-07-00-N | 39-40-00-E | 2698 |
| 79 | 3647 | EBL079 | 1979 | ETH | Oromia | BALE | S. DINSHO | Bale | 07-07-00-N | 39-40-00-E | 2555 |
| 80 | 3648 | EBL080 | 1979 | ETH | Oromia | BALE | S. DINSHO | Bale | 07-07-00-N | 39-40-00-E | 2986 |
| 81 | 3658 | EBL081 | 1979 | ETH | Oromia | BALE | S. DINSHO | Bale | 07-51-00-N | 39-38-00-E | 2400 |
| 82 | 3659 | EBL082 | 1979 | ETH | Oromia | BALE | S. DINSHO | Bale | 07-51-00-N | 39-38-00-E | 2400 |
| 83 | 3833 | EBL083 | 1979 | ETH | Oromia | BALE | S. DINSHO | Bale | 07-08-00-N | 40-00-00-E | 2530 |
| 84 | 4457 | EBL084 | 1980 | ETH | Oromia | BALE | RAYTU | Bale | 06-59-00-N | 39-11-00-E | 2677 |
| 85 | 4458 | EBL085 | 1980 | ETH | Oromia | BALE | RAYTU | Bale | 06-59-00-N | 39-11-00-E | 2287 |
| 86 | 4459 | EBL086 | 1980 | ETH | Oromia | BALE | RAYTU | Bale | 06-59-00-N | 39-11-00-E | 2623 |
| 87 | 4460 | EBL087 | 1980 | ETH | Oromia | BALE | RAYTU | Bale | 06-59-00-N | 39-11-00-E | 2545 |
| 88 | 4461 | EBL088 | 1980 | ETH | Oromia | BALE | RAYTU | Bale | 06-59-00-N | 39-11-00-E | 2829 |
| 89 | 4462 | EBL089 | 1980 | ETH | Oromia | BALE | RAYTU | Bale | 06-59-00-N | 39-11-00-E | 2632 |
| 90 | 4463 | EBL090 | 1980 | ETH | Oromia | BALE | RAYTU | Bale | 06-59-00-N | 39-11-00-E | 2709 |
| 91 | 4468 | EBL091 | 1980 | ETH | Oromia | BALE | RAYTU | Bale | 06-59-00-N | 39-11-00-E | 2899 |
| 92 | 4718 | EBL092 | 1979 |  | Oromia | BALE | S. DINSHO | Bale | 07-49-00-N | 39-58-00-E | 2470 |
| 93 | 64207 | EBL093 | 1981 | ETH | Oromia | BALE | ADABA | Bale | 07-02-00-N | 39-32-00-E | 2650 |
| 94 | 64208 | EBL094 | 1981 | ETH | Oromia | BALE | ADABA | Bale | 07-02-00-N | 40-32-00-E | 2650 |
| 95 | 64210 | EBL095 | 1981 | ETH | Oromia | BALE | ADABA | Bale | 07-05-00-N | 39-32-00-E | 3400 |
| 96 | 64211 | EBL096 | 1981 | ETH | Oromia | BALE | ADABA | Bale | 07-06-00-N | 39-32-00-E | 3400 |
| 97 | 64217 | EBL097 | 1981 | ETH | Oromia | BALE | S. DINISHO | Bale | 27-06-00-N | 39-48-00-E | 2950 |
| 98 | 64218 | EBL098 | 1981 | ETH | Oromia | BALE | S. DINSHO | Bale | 07-06-00-N | 39-48-00-E | 2950 |
| 99 | 64219 | EBL099 | 1981 | ETH | Oromia | BALE | S. DINSHO | Bale | 07-06-00-N | 39-48-00-E | 2950 |
| 100 | 64220 | EBL100 | 1981 | ETH | Oromia | BALE | S. DINSHO | Bale | 07-06-00-N | 39-48-00-E | 2950 |
| 101 | 64221 | EBL101 | 1981 | ETH | Oromia | BALE | S. DINSHO | Bale | 07-06-00-N | 39-48-00-E | 2810 |
| 102 | 64222 | EBL102 | 1981 | ETH | Oromia | BALE | S. DINSHO | Bale | 07-06-00-N | 39-48-00-E | 2800 |
| 103 | 64223 | EBL103 | 1981 | ETH | Oromia | BALE | GOBA | Bale | 07-13-00-N | 39-59-00-E | 2600 |
| 104 | 64224 | EBL104 | 1981 | ETH | Oromia | BALE | GOBA | Bale | 07-13-00-N | 39-59-00-E | 2600 |
| 105 | 64225 | EBL105 | 1981 | ETH | Oromia | BALE | GOBA | Bale | 07-03-00-N | 39-59-00-E | 2500 |
| 106 | 64226 | EBL106 | 1981 | ETH | Oromia | BALE | GOBA | Bale | 07-03-00-N | 39-59-00-E | 2500 |
| 107 | 64227 | EBL107 | 1981 | ETH | Oromia | BALE | S. DINSHO | Bale | 07-07-00-N | 40-02-00-E | 2450 |
| 108 | 64228 | EBL108 | 1981 | ETH | Oromia | BALE | S. DINSHO | Bale | 07-07-00-N | 40-02-00-E | 2450 |
| 109 | 64229 | EBL109 | 1981 | ETH | Oromia | BALE | S. DINSHO | Bale | 07-07-00-N | 40-02-00-E | 2450 |
| 110 | 64230 | EBL110 | 1981 | ETH | Oromia | BALE | S. DINSHO | Bale | 07-06-00-N | 40-44-00-E | 2470 |
| 111 | 64232 | EBL111 | 1981 | ETH | Oromia | BALE | S. DINSHO | Bale | 07-06-00-N | 40-49-00-E | 2410 |
| 112 | 64233 | EBL112 | 1981 | ETH | Oromia | BALE | S. DINSHO | Bale | 07-05-00-N | 40-14-00-E | 2410 |
| 113 | 64234 | EBL113 | 1981 | ETH | Oromia | BALE | S. DINSHO | Bale | 07-04-00-N | 40-14-00-E | 2460 |
| 114 | 64235 | EBL114 | 1981 | ETH | Oromia | BALE | S. DINSHO | Bale | 07-04-00-N | 40-14-00-E | 2450 |
| 115 | 64236 | EBL115 | 1981 | ETH | Oromia | BALE | S. DINSHO | Bale | 07-04-00-N | 40-14-00-E | 2400 |
| 116 | 64237 | EBL116 | 1981 | ETH | Oromia | BALE | S. DINSHO | Bale | 07-01-00-N | 40-20-00-E | 1950 |
| 117 | 64238 | EBL117 | 1981 | ETH | Oromia | BALE | S. DINSHO | Bale | 07-08-00-N | 40-00-00-E | 2410 |
| 118 | 64239 | EBL118 | 1981 | ETH | Oromia | BALE | S. DINSHO | Bale | 07-09-00-N | 41-00-00-E | 2370 |
| 119 | 64240 | EBL119 | 1981 | ETH | Oromia | BALE | S. DINSHO | Bale | 07-11-00-N | 40-02-00-E | 2400 |
| 120 | 64241 | EBL120 | 1981 | ETH | Oromia | BALE | S. DINSHO | Bale | 07-17-00-N | 40-06-00-E | 2400 |
| 121 | 64242 | EBL121 | 1981 | ETH | Oromia | E. BALE | G. GOLELCHA | Bale | 07-22-00-N | 40-16-00-E | 2400 |
| 122 | 64243 | EBL122 | 1981 | ETH | Oromia | E. BALE | G. GOLELCHA | Bale | 07-06-00-N | 39-51-00-E | 2430 |
| 123 | 64244 | EBL123 | 1981 | ETH | Oromia | E. BALE | G. GOLELCHA | Bale | 07-09-00-N | 39-51-00-E | 2510 |
| 124 | 64245 | EBL124 | 1981 | ETH | Oromia | E. BALE | G. GOLELCHA | Bale | 07-09-00-N | 39-51-00-E | 2510 |
| 125 | 204674 | EBL125 | 1982 | ETH | Oromia | E. BALE | G. GOLELCHA | Bale | 07-07-00-N | 39-52-00-E | 2730 |
| 126 | 208816 | EBL126 | 1984 | ETH | Oromia | BALE | GOBA | Bale | 07-07-00-N | 40-02-00-E | 2450 |
| 127 | 208817 | EBL127 | 1984 | ETH | Oromia | W.Arsi | ADABA | Bale | 07-03-00-N | 39-33-00-E | 3500 |
| 128 | 212826 | EBL128 | 1984 | ETH | Oromia | BALE | S. DINSHO | Bale | 07-04-00-N | 39-53-00-E | 2550 |
| 129 | 212840 | EBL129 | 1984 | ETH | Oromia | E. BALE | G. GOLELCHA | Bale | 07-22-00-N | 40-12-00-E | 2390 |
| 130 | 212841 | EBL130 | 1984 | ETH | Oromia | BALE | GOBA | Bale | 06-55-00-N | 39-53-00-E | 2820 |
| 131 | 212842 | EBL131 | 1984 | ETH | Oromia | BALE | GORO | Bale | 06-58-00-N | 40-32-00-E | 1750 |
| 132 | 212843 | EBL132 | 1984 | ETH | Oromia | BALE | GORO | Bale | 07-01-00-N | 40-27-00-E | 1900 |
| 133 | 212844 | EBL133 | 1984 | ETH | Oromia | BALE | GORO | Bale | 07-06-00-N | 40-12-00-E | 2030 |
| 134 | 212845 | EBL134 | 1984 | ETH | Oromia | E. BALE | G. GOLELCHA | Bale | 07-22-00-N | 40-12-00-E | 2400 |
| 135 | 212846 | EBL135 | 1984 | ETH | Oromia | BALE | DODOLA | Bale | 06-50-00-N | 39-05-00-E | 2730 |
| 136 | 212847 | EBL136 | 1984 | ETH | Oromia | BALE | NENSEBO | Bale | 06-35-00-N | 39-13-00-E | 2300 |
| 137 | 213495 | EBL137 | 1985 | ETH | Oromia | BALE | NENSEBO | Bale | 06-35-00-N | 40-13-00-E | 2229 |
| 138 | 215363 | EBL138 | 1985 | ETH | Oromia | BALE | GOBA | Bale | 07-05-00-N | 40-00-00-E | 2226 |
| 139 | 215364 | EBL139 | 1985 | ETH | Oromia | BALE | GOBA | Bale | 07-05-00-N | 40-00-00-E | 2344 |
| 140 | 215365 | EBL140 | 1985 | ETH | Oromia | BALE | S. DINSHO | Bale | 07-17-00-N | 40-06-00-E | 2420 |
| 141 | 215366 | EBL141 | 1985 | ETH | Oromia | BALE | S. DINSHO | Bale | 07-18-00-N | 40-06-00-E | 2420 |
| 142 | 215367 | EBL142 | 1985 | ETH | Oromia | BALE | S. DINSHO | Bale | 07-17-00-N | 40-07-00-E | 2410 |
| 143 | 215368 | EBL143 | 1985 | ETH | Oromia | BALE | S. DINSHO | Bale | 07-17-00-N | 40-06-00-E | 2420 |
| 144 | 215369 | EBL144 | 1985 | ETH | Oromia | BALE | S. DINSHO | Bale | 07-17-00-N | 41-06-00-E | 2400 |
| 145 | 215372 | EBL145 | 1985 | ETH | Oromia | BALE | S. DINSHO | Bale | 08-17-00-N | 40-06-00-E | 2390 |
| 146 | 215373 | EBL146 | 1985 | ETH | Oromia | BALE | S. DINSHO | Bale | 07-19-00-N | 40-07-00-E | 2390 |
| 147 | 215374 | EBL147 | 1985 | ETH | Oromia | BALE | S. DINSHO | Bale | 07-16-00-N | 42-06-00-E | 2530 |
| 148 | 215375 | EBL148 | 1985 | ETH | Oromia | BALE | S. DINSHO | Bale | 08-17-00-N | 40-06-00-E | 2550 |
| 149 | 215376 | EBL149 | 1985 | ETH | Oromia | BALE | S. DINSHO | Bale | 07-19-00-N | 40-06-00-E | 2888 |
| 150 | EBL150 | EBL150 | Local check | ETH | Oromia | BALE | SINANA | Bale | 07-16-00-N | 41-06-00-E | 2798 |

Keys: S. DINSHO = SINANA DINSHO; G. GOLOLCHA = GASERA GOLOLCHA; W. Arsi = West Arsi; E. Bale = East Bale; EBL = Ethiopian Barley Landraces
